# Statistical strategies for constructing health risk models with multiple pollutants and their interactions: possible choices and comparisons

**DOI:** 10.1186/1476-069X-12-85

**Published:** 2013-10-04

**Authors:** Zhichao Sun, Yebin Tao, Shi Li, Kelly K Ferguson, John D Meeker, Sung Kyun Park, Stuart A Batterman, Bhramar Mukherjee

**Affiliations:** 1 Department of Biostatistics, University of Michigan School of Public Health, Ann Arbor, MI USA; 2 Department of Environmental Health Sciences, University of Michigan School of Public Health, Ann Arbor, MI USA

**Keywords:** Bayesian model averaging, Classification and regression tree, Collinearity, Interaction effect, Model selection, Multiple pollutants, Principal component analysis, Shrinkage, Variable selection

## Abstract

**Background:**

As public awareness of consequences of environmental exposures has grown, estimating the adverse health effects due to simultaneous exposure to multiple pollutants is an important topic to explore. The challenges of evaluating the health impacts of environmental factors in a multipollutant model include, but are not limited to: identification of the most critical components of the pollutant mixture, examination of potential interaction effects, and attribution of health effects to individual pollutants in the presence of multicollinearity.

**Methods:**

In this paper, we reviewed five methods available in the statistical literature that are potentially helpful for constructing multipollutant models. We conducted a simulation study and presented two data examples to assess the performance of these methods on feature selection, effect estimation and interaction identification using both cross-sectional and time-series designs. We also proposed and evaluated a two-step strategy employing an initial screening by a tree-based method followed by further dimension reduction/variable selection by the aforementioned five approaches at the second step.

**Results:**

Among the five methods, least absolute shrinkage and selection operator regression performs well in general for identifying important exposures, but will yield biased estimates and slightly larger model dimension given many correlated candidate exposures and modest sample size. Bayesian model averaging, and supervised principal component analysis are also useful in variable selection when there is a moderately strong exposure-response association. Substantial improvements on reducing model dimension and identifying important variables have been observed for all the five statistical methods using the two-step modeling strategy when the number of candidate variables is large.

**Conclusions:**

There is no uniform dominance of one method across all simulation scenarios and all criteria. The performances differ according to the nature of the response variable, the sample size, the number of pollutants involved, and the strength of exposure-response association/interaction. However, the two-step modeling strategy proposed here is potentially applicable under a multipollutant framework with many covariates by taking advantage of both the screening feature of an initial tree-based method and dimension reduction/variable selection property of the subsequent method. The choice of the method should also depend on the goal of the study: risk prediction, effect estimation or screening for important predictors and their interactions.

## Background

As public awareness of consequences of environmental exposures has grown, estimating the adverse health effects due to simultaneous exposure to multiple pollutants is currently an important topic to explore [1]. It can provide insight into understanding the biological mechanisms of pollutant toxicity and guide regulatory standards for public health [2,3]. To date, numerous studies have examined a wide range of health impacts from exposure to ambient pollutants, with positive evidences on elevated all-cause mortality [4,5], impaired cardiac function [6], adverse cardiovascular events [7,8], propensity for diabetes mellitus [9], raised incidence of respiratory symptoms in children [10,11], reduced lung function [12,13], preterm delivery and low birth weight [14,15] and increased cancer risk [16,17].

In air pollution epidemiology, traditionally, health risks were assessed by estimated effects of one or several monitored pollutants using single-pollutant models. For instance, fine particulate matter (PM_2.5_) has been one of the most frequently studied pollutants [6,7,10,14,16,18-20]. Other criteria air pollutants such as carbon monoxide (CO), sulfur dioxide (SO_2_), nitrogen dioxide (NO_2_) and ozone (O_3_) also have been implicated in adverse effects on health [14,18,19]. However, the reliability of using single pollutant as the surrogate for air pollution is inadequate because air pollution is a mixture of many different gases, vapors and particles, with varying concentration and composition depending on the geographic regions and meteorological conditions [21]. To capture the complex nature of environmental exposure as a whole, there is a need to move from a single-pollutant to a multipollutant approach, as recommended by the National Research Council [1,21-24].

Some of the major challenges in evaluating the health impacts of environmental exposures in a multipollutant model are (1) to identify the specific components of pollution mixture that are most critical to the outcome of interest, when a large number of exposures are observed; (2) to examine the potential for interaction effects among pollutants, given the evidence that the impact of combined exposures may differ from the sum of the impacts from single-pollutant models [25]; and (3) to efficiently attribute health effects to individual pollutants in the presence of multicollinearity. Pollutants are often correlated due to the temporal and spatial pattern of emissions, and the strong influence of common meteorology. The various common constituents of the atmosphere and their chemical reactions across a region can induce this temporal and spatial correlation [26]. Additionally, multipollutant models place higher demands on the completeness and quality of the necessary exposure data, which has been a longstanding issue in environmental epidemiology [21]. While very important, the issue of exposure assessment and areal integration/imputation of possibly misaligned exposure data is beyond the scope of our coverage in the present paper.

Often, surrogates or proxy summary measures of the total environmental exposure burden have been used to explore the deleterious effects of pollution mixture. A simple way is to represent each emission source by a single pollutant, for instance, NO_2_ or CO for vehicular traffic, PM_10_ for traffic and other combustion sources, and SO_2_ as an indicator of power plant emissions. A second approach involves replacing ambient concentrations of pollutants with an indicator variable for the emission source. Factors such as living in close proximity to major roads or around roads with certain traffic intensity have been used to assess health risks [16]. A third strategy divides pollutants into groups by physical-chemical characteristics or biological reactivity. For example, the separation of fine (PM_2.5_) and coarse particles (PM_10-2.5_) based on size distribution has sharpened our understanding of where inhaled size-specific particulate matters come to rest in human body and how they lead to different biological symptoms [6,12]. A fourth strategy uses source apportionment analysis or “receptor modeling”, a widely used technique that distinguishes emission sources, especially for PM, by apportionment using profiles and source impacts [27]. A few epidemiological studies have used source apportionment data as explanatory variables in their examinations of PM health effects [28,29]. Lastly, composite metrics, such as the Air Quality Index (AQI) proposed by the U.S. Environmental Protection Agency (EPA), might be used to explore health impacts of air pollution. The AQI provides an integrated value of air quality health impacts from five air pollutants (O_3_, PM, CO, SO_2_, and NO_2_) regulated by the Clean Air Act, and is categorized into six levels labeled as “good”, “moderate”, “unhealthy for sensitive groups”, “unhealthy”, “very unhealthy” and “hazardous”, each accompanied with difference health advices [30]. While the strategies above could be easily implemented, overly simplified conversion to surrogate categories may lead to loss of information, and the resulting estimates corresponding to surrogates or proxies have distinct interpretations than the ones corresponding to individual exposures. More importantly, synergistic effects among pollutants become not identifiable by substituting with the composite or mixture measures.

To address potential synergistic effects among air pollutants, multivariate models with main effects for each pollutant and interaction effects for each pair of pollutants has been suggested [1,21]. However, it is well known that the statistical power for detecting a significant two-way interaction is low unless there is a strong measurable interaction or an adequately large sample size, and this reduction in power is even greater for higher order interactions. Consequently, it may not be feasible to detect all pairwise (and higher order) interactions given a large set of pollutants. When a synergy among multiple pollutants is suspected, tree-based regression methods such as classification and regression tree (CART) can be used to explore multiple potentially non-linear and hierarchical interactions [31]. While CART is well suited for identifying important main effects and interactions, it does not lead to quantitatively meaningful effect estimates. An alternative to quantify health effects with complex interactions is the deletion/substitution/addition (DSA) algorithm by Sinisi and van der Laan [32]. This algorithm was proposed to explore interactions in high-dimensional genomic data, and was later adapted to the environmental context to overcome the issue of a large number of correlated predictors [33]. One advantage of this algorithm is its flexibility of imposing customized constraints on model size, order of interactions, and polynomial functions of exposures to be included in the model. In addition, its model selection process is more aggressive than other automated selections as it does not enforce the restriction of nested structure on the explored class of models. However, the DSA approach has been criticized because its estimates are not consistent if the ratio of sample size to the number of candidate predictors is small, and the associated confidence intervals have poor statistical properties when substantial correlation exists among predictors [34].

To identify risk factors that have the most explanatory power in the presence of correlation, a natural route is to apply some dimension reduction technique. Several standard methods, such as factor analysis or principal component analysis (PCA), have been implemented to analyze the effects of multiple pollutants [35-37]. PCA derives orthogonal components from the set of exposure variables by making full use of the variability in the data by using the eigenvalues and eigenvectors of the exposure correlation matrix. A resultant “eigenexposure” can be used in subsequent analysis. A danger in this analysis is that the relationship between exposures and response variables is not accounted for in the generation of the principal components. A modified version of PCA, named supervised principal component analysis (SPCA), may go beyond this limit [38]. By a pre-screen of eliminating pollutants not associated with the outcome, SPCA returns effect estimates with smaller bias than corresponding estimates from PCA. However, after the pre-screen step, the limitation of not accounting for the multivariate exposure-response associations in PCA still applies to SPCA, and it may suffer from model misspecification. Partial least-squares regression (PLSR) is another extension of PCA, in which an optimum subset of predictors is found that is also relevant for response-predictor relationships [39-41]. Despite its popularity in engineering and machine learning community, PLSR has been seldom used in environmental health studies. One disadvantage of all PCA-based techniques is that since each principal component is a linear combination of multiple pollutants, estimated coefficients do not have direct quantitative interpretation.

While PCA offers dimension reduction in constructing summary exposure features, shrinkage-based regression, e.g., least absolute shrinkage and selection operator (LASSO) or ridge regression, may be helpful in dealing with large dimensional models through variable selection [42]. Due to the nature of the linear constraint, LASSO regression differs from ridge regression in that exact shrinkage of coefficients to zero is feasible, and hence it incorporates variable selection [43]. Unfortunately, shrinkage-based methods are unable to provide unbiased effect estimates, and some of them (i.e., ridge regression) do not perform variable selection.

A further statistical difficulty involves incorporating model uncertainty in the effect estimates. Given a long list of explanatory variables, the usual practice is to present results from a single model selected from a series of hypothesis testing procedures while ignoring other plausible models. Skepticism regarding this philosophy that a single model can serve as a representative of “the true model” in a given dataset with moderate sample sizes prevails [44]. To address this problem of selecting “the” model, Bayesian model averaging (BMA) averages the effect estimates across all possible models weighted by the model posterior probabilities [45,46]. However, there are differences in opinion associated with the interpretation of effect estimates from BMA in the presence of extensive collinearity or under a large model space [46].

From the above discussion it is clear that the choice of the method has to be guided by the goal of the study: screening for associations, effect estimation or risk prediction; it also has to be governed by the size of the dataset as well as the dimensionality of the potential set of predictors. There is a paucity of literature reviewing the operating characteristics of existing statistical models and comparing their strengths and weaknesses in a multipollutant context. Billionnet et al. recently presented an excellent review of statistical methods used or potentially applicable to this problem [47]. However, no quantified assessment of the relative performances of different methods or numerical results from simulation studies was provided in that review. In this paper, we conduct an extensive simulation study for continuous outcomes from a cross-sectional study as well as disease counts from a time-series study, and present two data examples to assess the performances of the five statistical methods: DSA, SPCA, LASSO, PLSR and BMA. In addition, a two-step modeling strategy employing an initial screening by CART in combination with each of the aforementioned five methods is evaluated when there is a long list of candidate pollutants plus interactions to consider. We examine the performance of each approach with a focus on feature selection, effect estimation and detection of main effects and interactions. Other aspects such as efficiency of the estimates, stability of the results, flexibility of implementation and difficulty in interpretation are discussed as well. This comprehensive comparison will be helpful for making informed decisions on which analytic approach to choose with different data structures and study designs. We also provide annotated R-codes for implementation of all the methods that may serve as a useful resource to the practitioner (see Additional file 1).

## Methods

### Statistical methods

In this section, we describe how each method is implemented and what are the advantages and disadvantages of using each one. All statistical analyses can be conducted using R software (version 2.13.0), and packages corresponding to different methods will be introduced.

### Notations

Let us consider a regression problem with a continuous response *Y* and potential predictors *X* (different pollutants or source components in a single pollutant in the context of a multipollutant study):

(1) $$\begin{array}{l} Y=\beta_{0}+\sum_{p=1}^{P}\beta_{p}Z_{p}+\epsilon ,\mathrm{and}\\ Z_{p}=\underset{K\;\mathrm{terms}}{\underset{︸}{X_{1},\ldots ,X_{K},}}\underset{(\begin{array}{c} K\\ 2 \end{array})\;\mathrm{terms}}{\underset{︸}{X_{1}X_{2},\ldots ,X_{K-1}X_{K}}},\mathrm{say}; \end{array}$$

where *β*_0_ refers to the intercept, *β*_*p*_ is the coefficient for predictor *Z*_*p*_ (can be zero in the true model), the random error variable *ϵ* follows a standard normal distribution, *X* is a set of candidate pollutants with size *K*, and *Z* is the entire set of potential predictors: for this paper we consider all the main effect terms and pairwise interactions among candidate pollutants in *X*, hence the dimension of *Z* is $P=K+\left({}_{2}^{K}\right)$.

### Classification and regression tree (CART)

The algorithm of CART involves recursively partitioning observations until reduction in the variability of the outcome is maximized [48]. The construction of a decision tree involves three steps [31,43]: (1) Recursive data partitioning: at each partitioning step, the algorithm examines all split points on every explanatory variable in *X*, and chooses the best pair of split points and splitting variable in terms of the minimum sum of squared deviations from the mean responses in the resultant two subgroups. This partitioning is repeated on each of the new subgroups. In this process, no assumption of linear relationship has been assumed, so complex interactions and non-linear effects may be captured. (2) Construction of the preliminary tree: in order to capture the important structure of the data, a large preliminary tree is preferred and the binary splitting is stopped when a minimum node size is reached or when further split does not improve the overall fit significantly. (3) Pruning the decision tree: to overcome the problem of overfitting, the preliminary tree is pruned by its predictive ability, for example, minimizing the ten-fold cross-validated error. This recursive partitioning of the regression trees can be implemented by *rpart* package [48].

Unlike traditional regression models, a tree-based method has several attractive properties: it is less sensitive to outliers, it requires no distributional assumption or data transformation, it is adaptable to complicated interactions among a large pool of predictors, the results are visually intuitive, and the prediction rule is easy to follow [38,41]. However, its application is restricted by the fact that quantified risk and effect estimates corresponding to the predictors cannot be obtained directly.

### Deletion/Substitution/Addition (DSA)

As a novel model selection approach, the implementation of DSA algorithm can be divided into three steps [32,49]: (1) Construct the whole model space as linear combinations of basis functions under user-specified constraints, where the choices of basis functions of candidate predictors are determined by the maximum order of interactions and maximum sum of powers (e.g. terms of the nature $X_{1}^{i}X_{2}^{j}$, with a constraint on *i+j*), and the model size also has a specified maximal value. (2) Starting from an intercept model, the DSA algorithm searches the model space by making deletion, substitution and addition moves repeatedly until the model size exceeds the specified maximum value. Given the current best model of size *p=3*, say (e.g., *X*_1_ + *X*_2_ + *X*_1_*X*_2_), a deletion move produces a deletion set of models with size *p-1* by deleting an existing term from the current model (e.g., *X*_1_ + *X*_2_), finds a model with the minimum objective function within this deletion set, and updates the best model of size *p-1* if this minimum is less than previously saved minimum of size *p-1*. The objective function chosen depends on the type of response considered and corresponds to sum of squared residuals in linear regression. The substitution and addition moves are performed similarly, with the substitution set containing models of the same size *p* where an existing term is replaced by a new term (e.g., *X*_1_ + *X*_2_ + *X*_1_*X*_3_) and the addition set containing models of size *p+1* by adding a new term to the current model (e.g., *X*_1_ + *X*_2_ + *X*_3_ + *X*_1_*X*_2_). (3) After the optimal model for each model size is identified, the final model with its corresponding predictors is selected based on cross-validation. This flexible model selection approach can be implemented by *modelUtils* and *DSA* package jointly [50].

Considering its original motivation of detecting transcription factor binding sites for the analysis of genomic data, the DSA algorithm was developed to enable an exhaustive search over the entire covariate space, which includes complex interactions and nonlinear terms of predictors, a feature that is likely to be useful in multipollutant studies. Another attraction of this algorithm is the adoption of the deletion, substitution and addition moves. Unlike automatic model selection such as backward or stepwise procedures which depend on tests for nested models, DSA allows for the flexibility of deleting, replacing or adding terms at each move, thus forcing the search to be more exhaustive. Additionally, the use of cross-validation in the algorithm ensures the selected model being less sensitive to outliers and has good predictive ability [33].

### Supervised principal component analysis (SPCA)

Acknowledging that conventional PCA only maximizes the variance explained by linear combinations of the predictor variables, SPCA was proposed to take into account the relationship between predictors and response variables in the dimension reduction process [51]. The benefit of SPCA as a feature selection tool becomes apparent when the covariate space grows, especially under extreme conditions where the number of covariates exceeds the number of observations, the well-known *P*>*N* situation.

Roberts and Martin further refined the SPCA method to make it suitable for multipollutant time-series studies [38]. For describing the method, we assume the regression dataset comes from a cross-sectional study with continuous responses as described in Equation 1. The implementation of SPCA can be achieved as follows: (1) Sort Wald’s statistics from univariate models for all individual and interaction terms in a descending order. (2) Choose a reduced matrix *Z’* selecting *S* covariates with absolute values of Wald’s statistics larger than a threshold *ζ*, where the optimal choice of *ζ* is determined by minimizing the prediction error of the corresponding multivariate model via 10-fold cross-validation. (3) Compute the first *q* (*q*<*S*) principal components of the reduced matrix *Z’*. Typically the first few principal components capture most of the variability in the covariate space, and in some cases it may be enough to use only the first principal component. (4) Refit the multipollutant model based on the linear combinations of *S* predictors retained in the reduced matrix *Z’*. As a result, the fitted model for SPCA can be explicitly expressed as:

(2) $$\mathrm{EY}=\sum_{i=1}^{q}b_{i}Q_{i}=\sum_{i=1}^{q}b_{i}\sum_{s=1}^{S}\alpha_{\mathrm{is}}Z{'}_{s}$$

where *Q*_*i*_ denotes the *i-th* principal component from the reduced matrix *Z’*, *b*_*i*_ is the specified effect of the *i-th* principal component, and *α*_*is*_ is the loading factor of the *s-th* predictor in matrix *Z’* for the *i-th* principal component. The *superpc* package provides SPCA analysis with continuous outcome [52]. Modified R codes as in Roberts and Martin [38] were used for implementation of SPCA in time-series studies with count data since no statistical packages are readily available.

By excluding covariates not strongly related to the response variables, SPCA reduces the bias of effect estimates in comparison with conventional PCA [38]. However, there are concerns on the loadings for predictors in the reduced matrix since they are computed without consideration of their multivariate associations with outcomes. Moreover, the interpretation of estimated coefficients from SPCA is difficult especially when more than one principal components of the reduced matrix are included.

### Least absolute shrinkage and selection operator (LASSO)

Shrinkage-based regression methods such as LASSO were developed to address the problem of fitting a regression model when the number of predictors is large compared to the sample size. By imposing a penalty on the size of the regression coefficients, they are expected to perform a bias-variance trade-off with the price of sacrificing unbiasedness of estimates for more precision [53]. The LASSO estimates are defined by minimizing the sum of squared errors with a bound on the sum of the absolute values of the coefficient estimates [43]:

(3) $${\hat{\beta }}_{\mathrm{LASSO}}=\underset{\beta }{\arg\;\mathrm{mi}}n\left\{\sum_{i=1}^{N}{\left(Y_{i}-\beta_{0}-\sum_{p=1}^{P}\beta_{p}Z_{\mathrm{ip}}\right)}^{2}+\lambda \sum_{p=1}^{P}\left|\beta_{p}\right|\right\}$$

where *N* denotes the sample size and *λ* is the tuning parameter that controls the amount of shrinkage. Due to the *L*_*1*_ penalty term, computation of the LASSO solutions is a quadratic programming problem and there is no closed form expression for estimates obtained from LASSO [53]. An efficient algorithm for all possible LASSO estimates using modified least angle regression (LAR) was proposed by Efron et al. [43,54]. Briefly, least angle regression iteratively builds models by including the predictor with the highest correlation to the current estimated residual. At each step, LASSO solutions are computed for a grid of shrinkage parameters, starting from zero to the least squares fit, and the optimal *λ* is selected by the minimum cross-validated root mean squared error. As a result, LASSO coefficient path for all predictors is constructed. The *lars* package was developed to implement this algorithm. Many existing prediction loss criteria, for example, the Mallows’ *Cp* statistic, can be used for selecting the optimal dimension of the LASSO model.

Despite a small bias introduced in the coefficients, LASSO regression has desirable analytical properties [55]. And by imposing a linear constraint, LASSO regression can shrink coefficients to exactly zero, a feature similar to variable selection that is not shared in ridge regression [56]. However, correct statistical inference following LASSO remains a challenging problem.

### Partial least-squares regression (PLSR)

PLSR is useful in constructing predictive models with high-dimensional covariates. Compared to PCA, PLSR often requires fewer components to achieve the same prediction error as it captures information in the predictors as well as the relationships between predictors and response variables [39,41]. There are several PLSR algorithms developed, among which a projection kernel based algorithm is used as the default method in the *pls* package in R and can be described through the following steps [40]: (1) Compute the first eigenvector of kernel matrices *Z*^*T*^*YY*^*T*^*Z* and *Y*^*T*^*ZZ*^*T*^*Y*, referred as weight vectors *l*_*1*_ and *h*_*1*_, respectively, and normalize both vectors so that ||*Zl*_*1*_|| = ||*Yh*_*1*_||= 1. (2) Project the matrix of covariates on its weights to get the *z*-scores *f*_*1*_ = *Zl*_*1*_. (3) Calculate loadings for covariates by ordinary least square (OLS) regression $r_{1}^{T}={\left(f_{1}^{T}f_{1}\right)}^{-1}f_{1}^{T}Z=f_{1}^{T}Z=l_{1}^{T}Z^{T}Z$. (4) Deflate the matrix of covariates $Z_{1}=Z-f_{1}r_{1}^{T}$. (5) Repeat steps 1–4 multiple times until all PLS components are determined. Thus, the PLSR coefficients can be estimated by $\hat{\beta }=L{\left(L^{T}Z^{T}\mathrm{ZL}\right)}^{-1}H^{T}$, where *L* and *H* denote matrices with the weight vectors *l*_*1*_ and *h*_*1*_ as their columns. The optimal number of components is chosen empirically by plotting the cross-validated predictive residual error sum of squares (PRESS) as a function of the number of components, and selecting the number of components that yields the first local minimum in PRESS statistic or by a pre-defined threshold on the sequential increments of the PRESS statistic.

A major limitation of the PLSR analysis is that as a PCA-based method, it is not always optimal in screening or removing predictors that have no association with the response, since its components are computed as linear combinations of all predictors. However, it still serves as a powerful dimension reduction and noise removal tool with the focus on prediction.

### Bayesian model averaging (BMA)

As an effective approach to deal with model uncertainty, BMA provides robust estimation of parameters by model averaging. Suppose Δ is the parameter of interest (coefficient corresponding to a particular predictor in *Z,* say), then its posterior distribution given all observations (*Y*, *Z*) can be expressed as an average of the posterior distributions of parameter Δ under each of the models considered, weighted by their posterior model probabilities [57,58]:

(4) $$P\left(\Delta |Y,Z\right)=\sum_{d=1}^{D}P\left(\Delta |M_{d},Y,Z\right)P\left(M_{d}|Y,Z\right)$$

where *M*_*1*_,…, *M*_*D*_ (*D* ≤ 2^P^) denote the models considered, and the posterior probability of model *M*_*d*_, according to Bayes’ rule, is given by:

(5) $$P\left(M_{d}|Y,Z\right)=\frac{P\left(Y|M_{d},Z\right)P\left(M_{d}\right)}{\sum_{i=1}^{D}P(Y|M_{i},Z)P\left(M_{i}\right)}$$

in which *P*(*M*_*d*_) denotes the prior probability of model *M*_*d*_ being the true model, and the marginal likelihood of model *M*_*d*_ can be computed by integrating the likelihood function *P*(*Y*|*θ*_*d*_, *M*_*d*_, *Z*) over the prior probability of model-specific parameter vector $\theta_{d}=\left(\beta_{d},\sigma_{d}^{2}\right)$, say.

(6) $$P\left(Y|M_{d},Z\right)=\int P\left(Y|\theta_{d},M_{d},Z\right)P\left(\theta_{d}|M_{d}\right)d\theta_{d}$$

The precise expression for the posterior mean and variance of Δ can be derived by employing the rule of conditional expectation and the law of total variance, respectively [59,60].

Estimation in BMA consists of three steps: (1) Choose the prior probability for each model and prior density of parameters in each model. (2) Compute the marginal likelihood and posterior model probability for each model considered. (3) Derive the weighted average posterior distribution of the parameters of interest, and estimate its associated mean and variance. It should be noted that during the implementation of BMA by *bicreg* function in the *bma* package, enumeration of all models will be computationally too expensive if the number of candidate predictors exceeds 30, and a preliminary model selection will be conducted by default, before the model averaging [61-63].

Compared to conventional modeling methods which ignore model uncertainty, BMA is attractive in that it does not select for a single “best” model and it makes inferences by averaging over a range of possible models. BMA-based confidence intervals are well-calibrated by taking account of both sampling variation within models and between-model uncertainty. One concern for the BMA analysis is the potential for large variance of estimates produced in the presence of extensive multicollinearity [46,64].

### Simulation studies

Health effects of environmental exposures have been examined widely in cross-sectional, time-series, cohort and case–control studies. In general, cross-sectional studies relate continuous (e.g. blood pressure, heart rate variability) or binary (e.g. acute asthma attacks) variables to exposures at a single time point [33,65], while time-series analyses associate the number of events (e.g. deaths, hospitalizations, or emergency visits) with changes in daily ambient level of pollutants/exposures [10]. In order to evaluate the performances of different model building/dimension reduction strategies, exposure-response relationships were simulated in this paper under two settings: continuous outcome from a cross-sectional study and daily event counts from a time-series study. Binary outcomes from a cross-sectional/cohort/case–control study can be analyzed in a similar fashion as continuous outcomes using a *logit* link function. Two hypothetical sets of candidate pollutants with different sizes (*K*=4 and *K*=10 or 20) were used to assess the effectiveness of statistical methods in terms of feature selection and estimation of regression coefficients involved in the health risk models. Therefore, a combination of four scenarios each with different subsets of nonzero main effects and interactions was investigated under each strategy: Scenarios 1 and 2 examine continuous responses in a cross-sectional study with 4 and 20 pollutants, respectively, and Scenarios 3 and 4 examine daily counts from a time-series study with 4 and 10 pollutants, respectively.

### Simulation settings for cross-sectional studies

Lognormal distribution is an empirically justified density for many pollutant concentration levels [66]. A multivariate structure with pairwise correlations instead of mutual independence allows for a better capture of multipollutant properties. Under the cross-sectional design with four pollutants, with *X*=(*X*_*1*_, *X*_*2*_, *X*_*3*_, *X*_*4*_) as a 4-pollutant random vector, exposure variables were generated from a multivariate lognormal distribution with the mean *μ*=E[*X*]=(1.20, 2.30, 1.89, 1.00) and the covariance matrix $\Sigma =E\left[\left(X-\mu \right){\left(X-\mu \right)}^{T}\right]=\left(\sigma_{\mathrm{ij}}^{2}\right)$, in which diagonal elements $\sigma_{\mathrm{ii}}^{2}$*=*1.00, *i*=1,…,4, and off-diagonal elements $\sigma_{12}^{2}$*=*0.52, $\sigma_{13}^{2}$ =0.35, $\sigma_{14}^{2}$ =0.28, $\sigma_{23}^{2}$ =0.57, $\sigma_{24}^{2}$ =0.54, and $\sigma_{34}^{2}$ =0.41. Here, the choice of distribution parameters was based on observed data on four common pollutants CO, NO_2_, PM_2.5_ and SO_2_ in the Detroit Asthma Morbidity, Air Quality and Traffic (DAMAT) study, where each of the pollutants were standardized to have a unit variance [10]. Similarly, in the simulation Scenario 2, we use *X* to denote a 20-component vector from a multivariate lognormal distribution with the mean *μ*=E[*X*]=(*μ*_*1*_,…,*μ*_*20*_), *μ*_*i*_=1.00, *i*=1,…,20, and the covariance matrix $\Sigma ={\left(\begin{array}{cc} \Sigma_{1} & 0_{5*15}\\ 0_{15*5} & \Sigma_{2} \end{array}\right)}_{20*20}$, where $\Sigma_{1}=\left(\sigma_{\mathrm{ij}}^{2}\right)$ with $\sigma_{\mathrm{ii}}^{2}$ =1.00, $\sigma_{\mathrm{ij}}^{2}$ =0.20, i=1,…,5, *j*=1,…,5, *i*≠*j*; and Σ_2_ denotes an identity matrix *I*_15*15_.

A normal linear regression model was used to generate the continuous outcome *Y* given *X* in the cross-sectional study. We considered the true generation model to be a standard multiple linear regression model including main effects of a subset of pollutants and some pairwise interactions, as follows:

(7) $$Y=\beta_{0}+\sum_{i=1}^{K}\beta_{i}X_{i}+\sum_{i=1}^{K}\sum_{j>i}^{K}\gamma_{\mathrm{ij}}X_{i}X_{j}+\epsilon$$

where *ϵ* was assumed to be independent and follow a normal distribution *ϵ* ~ *N*(0, 3^2^). Regression coefficients were pre-specified: in simulation Scenario 1 (*K*=4), *β*_0_ =0.1, *β*_2_= *β*_3_=0.5, *β*_*i*_=0, *i*=1,4, *γ*_*23*_=0.2, *γ*_*ij*_=0, (*i*, *j*)≠(2, 3); and in simulation Scenario 2 (*K*=20), *β*_0_=0.1, *β*_1_=*β*_2_=*β*_6_=*β*_7_=0.5, *β*_*i*_=0, *i*≠1, 2, 6 or 7, *γ*_*12*_=*γ*_*16*_ = *γ*_*67*_ =0.2, *γ*_*ij*_=0, (*i*, *j*)≠(1,2), (1,6) or (6,7). This choice of the error distribution and regression coefficients ensures that the ratio of the sum of squares for regression to the total sum of squares *R*^*2*^ was fixed at the level of 0.25. For each simulation scenario, we simulated 1000 datasets of a moderate sample size 250. No confounding factors were taken into account for the purpose of simplicity.

### Simulation settings for time-series studies

Under the time-series design with four pollutants, the multivariate vector *X*_*t*_ (*t*=1,…,400) corresponding to daily exposure measurements on a period of 400 days was generated by an autoregressive model depending on previous 10 days. Specifically,

(8) $$X_{t}=\left(\begin{array}{c} x_{1t}\\ x_{2t}\\ x_{3t}\\ x_{4t} \end{array}\right),X_{t}=c_{i}+\sum_{j=1}^{10}\left(\begin{array}{cccc} \phi_{1j} & 0 & 0 & 0\\ 0 & \phi_{2j} & 0 & 0\\ 0 & 0 & \phi_{3j} & 0\\ 0 & 0 & 0 & \phi_{4j} \end{array}\right)\cdot X_{t-j}+\mathrm{MVN}\left(0,\Sigma \right)$$

where *c*_*i*_ (*i*=1,…,4) denotes the constant vector controlling for the seasonal effect of *X*_*t*_, *ϕ*_*kj*_ is the partial autocorrelation function (PACF) coefficient for the *k*-*th* pollutant at lag day *j*, *k*=1,…,4 and *j*=1,…,10. Specification of seasonal effects and PACF coefficients were estimated from data in the DAMAT study [6] (see Additional file 2: Table S1). Here, we used the same set of means and covariance matrix corresponding to the four pollutants as in the simulation Scenario 1.

In time-series studies, daily counts as an outcome would be influenced by a number of factors, such as temporal trend or meteorological variation [35]. To eliminate these confounding factors, we adopted a method previously used to generate realistic time-series count data [38,67,68]. First, we fitted a Poisson regression with a log-linear link to the actual DAMAT data.

(9) $$\begin{array}{l} \log\left(\omega_{t}\right)=\beta_{\mathrm{int}}+\beta_{s}\mathrm{Seaso}n_{t}+\beta_{d}\mathrm{DO}W_{t}+\beta_{\mathrm{rh}}RH_{t}\\ \;+\beta_{\mathrm{temp}}\mathrm{Spline}\left(\mathrm{Tem}p_{t}\right)+\beta_{t}\mathrm{Spline}\left(t\right) \end{array}$$

In this model, *ω*_*t*_ refers to the expected number of asthma events on day *t*, the list of covariates includes season, day of the week (DOW), daily average relative humidity (RH), smooth function *Spline(.)* of daily average temperature (Temp) and day of the study (*t*). Next, the estimated counts of asthma events ${\hat{\omega }}_{t}$ were extracted and added as an offset to a pre-specified exposure-response relationship in a second Poisson regression:

(10) $$\log\left(\psi_{t}\right)=\log\left({\hat{\omega }}_{t}\right)+\sum_{i=1}^{K}\beta_{i}X_{\mathrm{it}}+\sum_{i=1}^{K}\sum_{j>i}^{K}\gamma_{\mathrm{ij}}X_{\mathrm{it}}X_{\mathrm{jt}}$$

where *ψ*_t_ refers to the time-varying mean counts (after removing the effect of the confounding factors), main and interaction effects of four pollutants were specified as *β*_1_=*β*_3_=0.3, *β*_i_=0, *i*≠1 or 3, *γ*_*13*_=0.1, *γ*_*ij*_=0, (*i*, *j*)≠(1, 3). Time-series asthma counts of length 400 days representing an average number of 13.3 counts/day were then generated from Poisson distributions with mean *ψ*_t_.

Time-series counts for simulation Scenario 4 were generated in a similar fashion, but assuming a ten-pollutant multivariate structure with mean *μ*=(*μ*_*1*_,…,*μ*_*10*_), *μ*_*i*_=1.00, *i*=1,…,10 and covariance matrix $\Sigma ={\left(\begin{array}{cc} \Sigma_{1} & 0_{4*6}\\ 0_{6*4} & \Sigma_{2} \end{array}\right)}_{10*10}$, where $\Sigma_{1}=\left(\sigma_{\mathrm{ij}}^{2}\right)$ and $\sigma_{\mathrm{ii}}^{2}$ =1.00, *i*=1,…,4, $\sigma_{12}^{2}$ =0.60, $\sigma_{13}^{2}$ =0.40, $\sigma_{14}^{2}$ =0.20, $\sigma_{23}^{2}$ =0.50, $\sigma_{24}^{2}$ =0.20, $\sigma_{34}^{2}$ =0.10 and Σ_2_ refers to an identity matrix *I*_6*6_. Seasonal effects were the same as in the four-pollutant framework, while PACF coefficients were obtained from the DAMAT data under an autoregressive model on previous 5 days for reduced complicity (see Additional file 2: Table S1). Parameters in the second Poisson regression model were specified as *β*_1_=*β*_3_=*β*_6_=*β*_9_= 0.2, *β*_i_=0, *i*≠1, 3, 6 or 9, *γ*_*13*_ = *γ*_*16*_ = 0.1, *γ*_*ij*_=0, (*i*, *j*)≠(1, 3) or (1, 6). Considering the increased number of pollutants involved, daily counts were simulated for 800 days with an average rate of 14.7 events/day. Although some time-series studies have assessed health effects of exposure to multiple pollutants on longer time scales, relative performances of different statistical methods should remain similar given the sample size we chose.

## Data analysis examples

### Cross-sectional design with quantitative outcome: oxidative stress biomarkers and environmental contaminant exposures in the national health and nutrition examination survey study

In addition to the simulation study, we applied the above methods to data from the National Health and Nutrition Examination Survey (NHANES) collected between 2005 and 2008. NHANES is an ongoing cross-sectional study designed to measure subject exposure to various environmental chemicals, dietary intake patterns, and various health outcomes [69]. Our previous studies indicated several associations between urinary phthalate metabolites and serum markers of oxidative stress in a large human population [70,71]. As a follow-up, this analysis examines the same association when phthalate exposure occurs in conjunction with exposure to other environmental contaminants that may also be capable of causing an oxidative stress response.

In this combined dataset, we included subjects aged 12 and up with complete data on all exposures, outcomes, and covariates, containing age, ethnicity, and poverty income ratio used in the sampling process, and gender, body mass index, serum cotinine, and urinary creatinine considered to be correlated with the outcome, which resulted in a final sample size of 3,773. The population distribution by covariates is presented in Additional file 2: Table S2 and a total of 25 exposures are categorized into 4 groups. The outcomes of interest were bilirubin and gamma-glutamyl transferase (GGT) which are believed to be systemic markers of oxidative stress. A conventional log-transformation was applied to all the exposures and outcomes. This study design corresponds to Scenario 2 in our simulation: cross-sectional data with continuous outcome and larger number of potential predictors. We did not incorporate the survey weights from NHANES in our analysis as many R packages for our methods of variable selection do not allow for adjustment for survey design. However, models were adjusted for some characteristics used in the creation of the survey weights, including age, ethnicity, and poverty income ratio.

The procedure to conduct our analysis can be summarized into six steps: (1) We first regressed the outcome (bilirubin or GGT) on the covariates listed above and used the residuals from the regression model as the response for model selection in the following steps. (2) We performed correlation analysis to assess the presence of collinearity within each group of environmental exposures (See Additional file 2: Table S3). When several exposures in the same group were highly correlated (Pearson correlation coefficient > 0.60), only the one with the smallest *p*-value in the single-exposure regression model was retained (see Additional file 2: Table S4). (3) We applied CART with the criteria of minimizing the cross-validated error to the reduced subset of exposures after the correlation analysis. Among a reduced number of 18 candidate exposures from Step 2, seven were selected in the construction of the regression tree for the outcome bilirubin, and eight were selected for the outcome GGT (See Additional file 2: Figure S5). (4) We applied our methods to the main effects and all pairwise interactions of individual exposures selected by CART, including BMA, LASSO, SPCA and DSA. (5) All predictors identified by different methods were incorporated into a bigger omnibus linear regression model, where the outcome was bilirubin or GGT and all covariates were controlled for. (6) Eventually, proposed models for bilirubin or GGT were constructed by eliminating non-significant predictors (p-value > 0.05) from the omnibus models and exposure effects were estimated using ordinary linear regression. Note that proper inference and testing after model selection is an incredibly challenging problem, and fitting standard models to yield measures of significance post-model selection obviously leads to inflated Type 1 error rates and overly optimistic results. The results from the presented model should thus be interpreted with this caveat in mind.

### Time-series design with count data: asthma morbidly and ambient air pollutants in the detroit asthma morbidity, air quality and traffic study

We applied the proposed methods to the Detroit Asthma Morbidity, Air Quality and Traffic (DAMAT) study. Li et al. (2011) analyzed time-series count data from 2004–2006 on asthma morbidity in the pediatric Medicaid population of Detroit, Michigan. Concentrations of pollutants PM_2.5_, CO, NO_2_ and SO_2_ were examined for potential associations. Statistically significant associations at 5-day lag and 3- and 5-day moving averages of SO_2_ and PM_2.5_ concentrations were observed with asthma emergency department visits and hospitalizations using single-pollutant models [10]. We now extend our scope to multipollutant models exploring potential interactions to address the same research question.

Daily asthma events were identified from emergency department visits and hospitalizations for the Detroit Medicaid-insured population, and further restricted to children 2–18 years of age due to the difficulty of asthma diagnosis for children under 2. A total of 12,933 asthma events were observed from 7,063 children during the 1,096 days, representing an average rate of 11.8 events per day [10]. Daily measurements of CO, NO_2_, SO_2_ and PM_2.5_, and meteorological data including temperature and relative humidity, were also obtained for this study period. Previous analyses suggested strong evidence of rise in daily asthma events with increasing 3-day lag of concentrations for SO_2_ and PM_2.5_, so this lag specification of air pollutants was used as exposure variables. The mean 3-day lag of air pollutants were 0.43 ppm for CO, 16.8 ppb for NO_2_, 3.78 ppb for SO_2_, and 15.0 μg/m^3^ for PM_2.5_. This study design corresponds to Scenario 3 in our simulation analyses: time-series data with a count response and four air pollutants measured at 3-day lags.

To control for the temporal pattern and weather effects in the time-series data, we applied the two-stage generalized linear model (GLM) as described in the data generation of Scenario 3 in the simulation. The estimated daily counts from the first Poisson regression in (12) were used as an offset in the second Poisson regression model in (13) where the *X’*s represent the four exposures. Note that this two-step approach is adopted only due to a logistical inconvenience that some of the model selection tools available in existing statistical software do not allow inclusion of a set of “forced variables” in the model without performing variable selection on this set of confounders. Ideally, one would work with a single model that performs variable selection in the presence of confounders to be adjusted for.

The collection of main effects and pairwise interactions identified to be associated with the outcome within each method were incorporated into an omnibus GLM, with confounding factors such as year, season, DOW, time, RH and Temp adjusted and potential over-dispersion considered. Finally, a proposed model with non-significant exposure variables eliminated was constructed.

## Results

### Simulation results

Under each simulation configuration, we generated 1000 datasets. For each examined study design, we present the average of estimated effects with their empirical standard errors for non-zero predictors, the percentage of models correctly identifying the non-zero coefficients, and the average model size over 1000 replicates for five different statistical methods (BMA, DSA, LASSO, PLSR and SPCA). We also considered additional measures, including false positive rate (FPR), true positive rate (TPR), and mean squared error (MSE) for the coefficients corresponding to truly null and truly associated predictors, to help evaluate the performances of competing statistical methods as displayed in Additional file 2: Table S6.

For the ease of interpretation, the regression model in SPCA was fit on the first principal component of the reduced matrix Z’, and accordingly the estimated effects of predictors highly associated with the outcome can be expressed as the products of estimated regression coefficient for the first principal component b_1_ and the loading factors of predictors in the construction of first principal component α_1s_ (i.e. ${\hat{\beta }}_{s}=b_{1}\alpha_{1s}$ if *Z*_*s*_ ∈ *Z* '). In BMA analysis, predictors were considered to be identified if their posterior probabilities exceeded 10%, where the cut-off value was chosen in an ad-hoc manner. In the presence of a long list of candidate pollutants, we also assessed identification and estimation of non-zero coefficients using a two-step modeling strategy, which employs an initial screening by CART followed by further dimension reduction/variable selection by the five methods at the second step. This strategy was not used in the time-series studies because existing package does not apply CART to count data. The main findings of the simulation study are summarized as follows.

### Cross-sectional studies

Table 1 presents simulation results corresponding to the five methods under simulation Scenario 1 (cross-sectional design, *K*=4). Note that LASSO estimates of non-zero coefficients are the least biased and most efficient, with smallest MSE among all methods as displayed in Additional file 2: Table S6. Point estimates of interaction effects from PCA-based approaches such as SPCA and PLSR stay close to the true parameters, whereas substantial bias has been observed in their estimated main effects. Main effects from DSA are overestimated, while in BMA analysis the main effects are underestimated and interaction effect overestimated. These directions of upward or downward bias are not consistent across simulation settings. In PLSR, each component is constructed as a weighted mean of all candidate predictors, so all predictors are used in the regression model with its model size equal to the maximum model size. LASSO regression, SPCA and BMA have high interaction detection rates as assessed by the percentage of correct identification of the interaction term, while DSA algorithm barely identifies interaction with a very low rate of 4.4%. In terms of variable selection, BMA and LASSO regression are preferred with lower FPRs, high TPRs, and average model sizes close to the true one. SPCA tends to select a larger model as represented by its average model size and high FPR.

**Table 1:** Simulation results comparing five statistical methods under Scenario 1

**Predictor**	**β**	**Measure**	**BMA**^**1**^	**DSA**^**2**^	**LASSO**^**3**^	**PLSR**^**4**^	**SPCA**^**5**^
*X*_*2*_	0.50	Estimate (ESE)	0.22 (0.40)	0.76 (0.62)	0.40 (0.39)	0.04 (0.02)	0.08 (0.18)
		Percent included	51.8%	N/A	70.8%	N/A	90.6%
*X*_*3*_	0.50	Estimate (ESE)	0.25 (0.44)	0.86 (0.59)	0.43 (0.43)	0.05 (0.03)	0.08 (0.18)
		Percent included	53.0%	N/A	67.9%	N/A	90.6%
*X*_2_*X_*3*_	0.20	Estimate (ESE)	0.29 (0.11)	0.02 (0.11)	0.19 (0.14)	0.23 (0.11)	0.16 (0.11)
		Percent included	96.0%	4.4%	83.2%	N/A	82.5%
		Average model size	3.2	4.5	3.7	10	7.1

Average estimated effects, empirical standard errors, percentage of correct identification of non-zero coefficients, and average model size corresponding to 5 statistical methods in a cross-sectional study with continuous responses and 4 candidate air pollutants. Sample size for each replicate was *N*=250. The true model size was 3 without accounting for the intercept, and the possible maximum model size was 10. ESE, empirical standard error of the estimate. Results are based on 1000 replicates.

Estimate of the non-zero predictor is calculated as the mean of the products that estimated regression coefficient of this predictor multiplies the indicator function that this predictor is correctly identified during each replication. The percentage of the non-zero predictor quantifies the proportion of correct identification of this predictor over 1000 replicates in each method. ^1^In BMA, predictors with their posterior probabilities greater than 10% are regarded as identified. ^2^In DSA, there is no variable selection for main effects as individual exposures are enforced when their interactions are of interest. Identification of interaction refers to the inclusion of interaction term in the cross-validated best predictive model. ^3^Predictors with their estimated LASSO regression coefficients not equal to zero are considered identified. ^4^No variable selection has been applied in PLSR because it uses all predictors. ^5^In SPCA, predictors are identified if their Wald’s statistics from univariate models are larger than a threshold value.

Table 2(A) displays simulation results obtained from four modeling approaches DSA, LASSO, PLSR and SPCA under simulation Scenario 2. As the number of exposure variables increases from 4 to 20, the total number of candidate predictors (210), including main effects (20) and all pairwise interactions (190), grows in a quadratic manner. In this setting, BMA results are not provided because the total number of explanatory variables exceeds the maximum limit 30 set by *bma* package, and a stepwise procedure is applied automatically to eliminate the redundant explanatory variables. Among four available approaches, none of these methods appear to be a desirable choice in terms of variable selection because an appreciable number of null predictors are included conservatively, but LASSO regression is much superior to others. Compared to the results from simulation Scenario 1, estimated main effects from LASSO regression are shrunk heavily in order to induce a sparse model and compensate for the instability caused by multicollinearity. Interestingly, estimation for interaction effects by LASSO regression appears to be robust, and its interaction detection rate remains high. DSA algorithm also selects small models, yet it has a very limited ability of identifying interactions. SPCA has high detection rates for all non-zero coefficients, but the use of this method is restricted by its considerably large FPR and average model size. The MSE of null predictors within PCA-based methods (i.e. SPCA and PLSR) has been found much smaller and this may relate to the weak associations between null predictors and the outcome considered in the construction of principal components (Additional file 2: Table S6).

**Table 2:** Simulation results under Scenario 2: single step versus two-step strategy

**Predictor**	***β***	**Measure**	**(A) One-step regression using all predictors**				**(B) Two-step strategy employing CART at screening step**				
			**DSA**	**LASSO**	**PLSR**	**SPCA**	**BMA**^**1**^	**DSA**^**2**^	**LASSO**^**3**^	**PLSR**^**4**^	**SPCA**^**5**^
*X*_*1*_	0.50	Estimate (ESE)	0.93 (0.29)	0.08 (0.19)	0.03 (0.01)	0.03 (0.04)	0.32 (0.38)	0.93 (0.29)	0.35 (0.36)	0.11 (0.04)	2.3×10^-4^ (0.01)
		Percent included	N/A	28.2%	N/A	98.5%	65.2%	N/A	68.3%	N/A	58.8%
*X*_*2*_	0.50	Estimate (ESE)	0.75 (0.27)	0.07 (0.22)	0.02 (0.01)	0.02 (0.03)	0.25 (0.32)	0.74 (0.25)	0.33 (0.38)	0.09 (0.04)	−2.7×10^-4^ (0.01)
		Percent included	N/A	22.6%	N/A	94.0%	63.5%	N/A	63.8%	N/A	58.9%
*X*_*6*_	0.50	Estimate (ESE)	0.88 (0.29)	0.07 (0.19)	0.03 (0.02)	0.02 (0.03)	0.29 (0.36)	0.88 (0.25)	0.36 (0.36)	0.10 (0.05)	−1.2×10^-4^ (0.01)
		Percent included	N/A	25.8%	N/A	96.2%	63.6%	N/A	67.4%	N/A	57.9%
*X*_*7*_	0.50	Estimate (ESE)	0.71 (0.26)	0.04 (0.22)	0.02 (0.01)	0.01 (0.02)	0.24 (0.30)	0.67 (0.26)	0.32 (0.34)	0.08 (0.04)	9.1×10^-4^ (0.01)
		Percent included	N/A	18.1%	N/A	82.4%	65.6%	N/A	64.3%	N/A	57.8%
*X*_1_*X_*2*_	0.20	Estimate (ESE)	0.002 (0.03)	0.17 (0.14)	0.07 (0.04)	0.07 (0.07)	0.24 (0.22)	0.006 (0.06)	0.21 (0.18)	0.27 (0.08)	0.28 (0.13)
		Percent included	0.3%	79.2%	N/A	96.3%	78.4%	1.1%	84.0%	N/A	98.6%
*X*_1_*X_*6*_	0.20	Estimate (ESE)	0.003 (0.05)	0.20 (0.18)	0.06 (0.03)	0.05 (0.06)	0.21 (0.26)	0.006 (0.08)	0.22 (0.22)	0.23 (0.08)	0.19 (0.11)
		Percent included	0.3%	77.3%	N/A	99.0%	66.7%	0.9%	78.1%	N/A	99.8%
*X*_6_*X_*7*_	0.20	Estimate (ESE)	0.002 (0.04)	0.17 (0.16)	0.06 (0.03)	0.03 (0.05)	0.25 (0.27)	0.004 (0.05)	0.21 (0.21)	0.19 (0.08)	0.15 (0.11)
		Percent included	0.3%	74.4%	N/A	94.1%	73.3%	0.5%	76.9%	N/A	98.6%
		Average model size	20.1	22.8	210	79.3	6.0	4.2	6.7	10.0	8.2

Average estimated effects, empirical standard errors, percentages of correct identification of non-zero coefficients, and average model size corresponding to four available statistical methods in a cross-sectional study with continuous responses and 20 air pollutants were provide in panel A. Similar results of five statistical methods after an initial CART variable selection using the two-step modeling strategy were summarized in panel B. Sample size for each replicate was *N*=250. The true model size was 7 without accounting for the intercept, and the possible maximum model size was 210. ESE, empirical standard error of the estimate. Results are based on 1000 replicates.

Estimate of the non-zero predictor is calculated as the mean of the products that estimated regression coefficient of this predictor multiplies the indicator function that this predictor is correctly identified during each replication. The percentage of the non-zero predictor quantifies the proportion of correct identification of this predictor over 1000 replicates in each method. ^1^In BMA, predictors with their posterior probabilities greater than 10% are regarded as identified. ^2^In DSA, there is no variable selection for main effects as individual exposures are enforced when their interactions are of interest. Identification of interaction refers to the inclusion of interaction term in the cross-validated best predictive model. ^3^Predictors with their estimated LASSO regression coefficients not equal to zero are considered identified. ^4^No variable selection has been applied in PLSR because it uses all predictors. ^5^In SPCA, predictors are identified if their Wald’s statistics from univariate models are larger than a threshold value.

CART is widely used for detecting complex effects among a large number of explanatory variables, so as an alternative strategy we decided to apply CART to the complete set of candidate predictors prior to the five regression methods. The simulation results from this two-step strategy are given in Table 2(B). In the initial step of construction of the decision tree with the minimum cross-validated error, 4 pollutants are correctly identified to be associated with the outcome over 1000 datasets with percentages greater than 60% (*X*_*1*_: 86.8%, *X*_*2*_: 70.8%, *X*_*6*_: 83.7%, and *X*_*7*_: 61.6%), whereas other pollutants less than 13%. This result suggests that as an exploratory procedure for variable selection, CART is able to detect important variables and reduce the number of variables for regression model. Compared to the simulation results in Table 2(A), there are substantial improvements on the variable selection for all approaches, as reflected by the appreciable reduction in average fitted model size and FPR. BMA analysis becomes feasible after initial screening by CART, and they perform similarly as in simulation Scenario 1. The bias of estimates of non-zero coefficients and the MSE of null predictors in LASSO regression are significantly reduced as expected, whereas estimates with their empirical standard errors and interaction detection rates in DSA algorithm do not change appreciably.

### Time-series studies

Table 3 compares performances of regression models under a time-series design with count data as response and 4 individual pollutants and their pairwise interactions as candidate variables. The implementation of the DSA algorithm is not available for time-series data. In contrast to the simulated cross-sectional studies with dominance of a few methods, BMA, LASSO and PLSR all appear to perform quite reasonably in terms of estimation of risk ratio coefficients as presented by their small biases and MSEs in Additional file 2: Table S6. Among four approaches, LASSO regression with its 100 percent detection rate for all individual and interaction terms provides least biased and most efficient estimates. BMA has a comparable performance as LASSO in terms of variable selection but produces less efficient estimates as displayed by larger empirical standard errors and MSEs. PLSR also provides estimates with small bias but does not have the feature of variable selection. In spite of the inconsistent estimates, the high interaction detection rate in SPCA indicates its potential value under this simulation scenario.

**Table 3:** Simulation results for four statistical methods under Scenario 3

**Predictor**	***β***	**Measure**	**BMA**^**1**^	**LASSO**^**2**^	**PLSR**^**3**^	**SPCA**^**4**^
*X*_*1*_	0.30	Estimate (ESE)	0.26 (0.18)	0.27 (0.05)	0.24 (0.07)	0.0013 (0.0070)
		Percent included	88.5%	100%	N/A	5.6%
*X*_*3*_	0.30	Estimate (ESE)	0.28 (0.15)	0.27 (0.04)	0.23 (0.06)	0.0005 (0.0036)
		Percent included	95.8%	100%	N/A	3.8%
*X*_1_*X_*3*_	0.10	Estimate (ESE)	0.11 (0.06)	0.11 (0.01)	0.10 (0.02)	0.19 (0.04)
		Percent included	97.7%	100%	N/A	100%
		Average model size	4.5	5.4	10	1.3

Average estimated effects, empirical standard errors, percentages of correct identification of non-zero coefficients, and average model size corresponding to four statistical methods in a time-series study with count response and 4 air pollutants. Sample size for each replicate was *N*=400. The true model size was 3 with intercept not counted, and the possible maximum model size was 10. ESE, empirical standard error of the estimate. Results are based on 1000 replicates.

Estimate of the non-zero predictor is calculated as the mean of the products that estimated regression coefficient of this predictor multiplies the indicator function that this predictor is correctly identified during each replication. The percentage of the non-zero predictor quantifies the proportion of correct identification of this predictor over 1000 replicates in each method. ^1^In BMA, predictors with their posterior probabilities greater than 10% are regarded as identified. ^2^Predictors with their estimated LASSO regression coefficients not equal to zero are considered identified. ^3^No variable selection has been applied in PLSR because it uses all predictors. ^4^In SPCA, predictors are identified if their Wald’s statistics from univariate models are larger than a threshold value.

A large number of candidate predictors in a time-series setting can be computationally intensive, so we simulated daily counts with 10 air pollutants and present results regarding relative performances of four approaches in Table 4. With an increased sample size, BMA becomes favorable in terms of effect estimation and variable selection. In comparison, LASSO regression yields less parsimonious models, but provides the smallest MSEs for both null and associated predictors. The estimated main effects by PCA-based methods PLSR and SPCA are not very informative, yet their estimations for interaction terms have small biases and the interaction detection rate in SPCA is high.

**Table 4:** Simulation results of four statistical methods under Scenario 4

**Predictor**	**β**	**Measure**	**BMA**^**1**^	**LASSO**^**2**^	**PLSR**^**3**^	**SPCA**^**4**^
*X*_*1*_	0.20	Estimate (ESE)	0.19 (0.12)	0.15 (0.04)	0.15 (0.03)	0.006 (0.009)
		Percent included	89.3%	99.9%	N/A	44.1%
*X*_*3*_	0.20	Estimate (ESE)	0.19 (0.09)	0.15 (0.03)	0.12 (0.03)	0.004 (0.006)
		Percent included	94.6%	99.8%	N/A	32.8%
*X*_*6*_	0.20	Estimate (ESE)	0.19 (0.09)	0.14 (0.03)	0.12 (0.04)	0.0006 (0.0014)
		Percent included	95.8%	99.9%	N/A	18.2%
*X*_*9*_	0.20	Estimate (ESE)	0.20 (0.09)	0.14 (0.03)	0.08 (0.03)	0.0001 (0.0006)
		Percent included	94.5%	99.9%	N/A	6.2%
*X*_1_*X_*3*_	0.10	Estimate (ESE)	0.10 (0.03)	0.11 (0.01)	0.10 (0.01)	0.10 (0.07)
		Percent included	99.2%	100%	N/A	97.1%
*X*_1_*X_*6*_	0.10	Estimate (ESE)	0.10 (0.03)	0.11 (0.01)	0.10 (0.01)	0.06 (0.05)
		Percent included	99.5%	100%	N/A	87.0%
		Average model size	13.1	21.1	55	9.8

Average estimated effects, empirical standard errors, percentages of correct identification of non-zero coefficients, and average model size corresponding to four statistical approaches in a time-series study with count response and 10 air pollutants. Sample size for each replicate was *N*=800. The true model size was 6 with intercept not counted, and the possible maximum model size was 55. ESE, empirical standard error of the estimate. Results are based on 1000 replicates.

Estimate of the non-zero predictor is calculated as the mean of the products that estimated regression coefficient of this predictor multiplies the indicator function that this predictor is correctly identified during each replication. The percentage of the non-zero predictor quantifies the proportion of correct identification of this predictor over 1000 replicates in each method. ^1^In BMA, predictors with their posterior probabilities greater than 10% are regarded as identified. ^2^Predictors with their estimated LASSO regression coefficients not equal to zero are considered identified. ^3^No variable selection has been applied in PLSR because it uses all predictors. ^4^In SPCA, predictors are identified if their Wald’s statistics from univariate models are larger than a threshold value.

## Results for data examples

### NHANES data on oxidative stress biomarkers and environmental contaminant exposures

Table 5 presents the results of model selection from different methods for the NHANES data. Predictors and their estimated effects from the proposed models are shown in Additional file 2: Table S7. For the outcome bilirubin, where the associations between bilirubin and multiple exposures are moderate, individual exposures methyl paraben (EPAR), mono (2-ethyl-5-oxohexyl) phthalate (MEOHP), perchlorate (P8) and triclosan (TCS) are statistically significant in the omnibus regression model. Among all the cross products selected by different methods, the interaction of TCS and EPAR is the only one significant in the omnibus model, and none of the methods allows a correct identification of this effect except for LASSO regression. Likely due to the strong associations between the outcome GGT and exposures, all individual exposures identified by CART are tested to be significant in the omnibus model. Among significant interactions indicated in this model, cross products of EPAR*propyl paraben (PPAR), EPAR*mono (3-carboxypropyl) phthalate (MCPP), and EPAR*mono-isobutyl phthalate (MiBP) are commonly selected in the four competing statistical methods, while propyl paraben*2,4,5-trichlorophenol (PPAR*2,4,5-TCP) and P8*mono(3-carboxypropyl) phthalate (MCPP) are selected only once.

**Table 5:** Results of model selection for the NHANES data (2005–2008)

**Method**	**Response variable - Bilirubin**		**Response variable - GGT**	
	**Main effects**	**Interactions**	**Main effects**	**Interactions**
BMA	OP, EPAR, P8, MEOHP, MiBP	OP*EPAR, OP*MEOHP, P8*MEOHP, MEOHP*MiBP	EPAR, PPAR, MCPP, MEOHP, MiBP, 2,5-DCP	EPAR*PPAR, EPAR*MCPP, EPAR*MiBP, MEOHP*2,5-DCP,
LASSO	OP, TCS, EPAR, P8, MCPP, MEOHP, MiBP	OP*EPAR, OP*MEOHP, TCS*EPAR, TCS*MCPP, EPAR*MCPP, P8*MCPP	EPAR, PPAR, P8, MCPP, MEOHP, MiBP, 2,5-DCP, 2,4,5-TCP	EPAR*PPAR, EPAR*P8, EPAR*MCPP, EPAR*MiBP, EPAR*2,5-DCP, PPAR*2,4,5-TCP, P8*MCPP, MCPP*2,4,5-TCP, MiBP*2,4,5-TCP
SPCA	OP, EPAR, P8, MCPP, MEOHP, MiBP	OP*P8, OP*MEOHP, P8*MCPP, P8*MEOHP, P8*MiBP	EPAR, PPAR, P8, MCPP, MEOHP, MiBP, 2,5-DCP, 2,4,5-TCP	EPAR*PPAR, MCPP*2,5-DCP, EPAR*MEOHP, EPAR*MiBP, EPAR*2,5-DCP, P8*MEOHP, MEOHP*2,5-DCP, P8*2,5-DCP, EPAR*P8
DSA	OP, TCS, EPAR, P8, MCPP, MEOHP, MiBP	N/A	EPAR, PPAR, P8, MCPP, MEOHP, MiBP, 2,5-DCP, 2,4,5-TCP	N/A

Phthalates: MEHP, mono(2-ethylhexyl) phthalate; MEHHP, mono(2-ethyl-5-hydroxyhexyl) phthalate; MEOHP, mono(2-ethyl-5-oxohexyl) phthalate; MECPP, mono(2-ethyl-5-carboxypentyl) phthalate; MnBP, mono-n-butyl phthalate; MiBP, mono-isobutyl phthalate; MBzP, mono-benzyl phthalate; MEP, mono-ethyl phthalate; MCPP, mono(3-carboxypropyl) phthalate. Phenols: BPA, bisphenol-A; TCS, triclosan; BPAR, butyl paraben; EPAR, ethyl paraben; MPAR, methyl paraben; PPAR, propyl paraben; BP3, benzophenone-3; OP, 4-tert octylphenol. Pesticides: 2,5-DCP, 2,5-dichlorophenol; 2,4-DCP, 2,4-dichlorophenol; OPP, o-phenyl phenol; 2,4,5-TCP, 2,4,5-trichlorophenol; 2,4,6-TCP, 2,4,6-trichlorophenol. Perchlorate and related anions: P8, perchlorate; NO3, nitrate; SCN, thiocyanate.

Examining results from the NHANES data suggest the usefulness of CART in feature selection. LASSO regression identifies all the significant main effects and interactions in the omnibus models but yields slightly large models. BMA and SPCA detect highly significant interactions (*p*≤0.001) but would miss a few weaker associations. For instance, among five significant interactions in the omnibus model for outcome GGT, the most significant two or three were typically detected by BMA and SPCA. Similar as in the simulation, DSA performs poorly by failing to detect any interactions either for bilirubin or GGT.

### DAMAT data on asthma morbidly and ambient air pollutants

Predictors identified from different methods for DAMAT data are displayed in Table 6, with their estimated effects in the omnibus model shown in Additional file 2: Table S8. Consistent with the simulation study and analysis of NHANES data, LASSO regression chooses the largest model among all methods. Although the performances of BMA and SPCA are slightly different, both of them are able to identify the most significant predictor PM_2.5_ in the proposed model but fail to detect the less significant effect of CO. No interactions were selected in the DAMAT study.

**Table 6:** Results of model selection for the DAMAT data (2004–2006)

**Method**	**Main effects**	**Interactions**
BMA	PM_2.5_	PM_2.5_*SO_2_
LASSO	CO, PM_2.5_, SO_2_	NO_2_*PM_2.5_, PM_2.5_*SO_2_
SPCA	PM_2.5_, SO_2_	PM_2.5_*SO_2_, NO_2_*SO_2_

## Discussion

This paper provides empirical guidance for selecting methods readily available in statistical packages to assess the health effects of environmental exposures in a multipollutant framework under conventional cross-sectional and time-series studies. A summary chart of all the methods discussed in this paper with corresponding references to the implementation software is presented in Table 7.

**Table 7:** Glossary of methods with implementation software

**Method**	**Reference type**	**Reference**	**Software**
Bayesian model averaging (BMA)	Theory	Madigan and Raftery, 1994 [58]	*bma* package in R
	Application	Koop and Tole, 2004 [45]	
Deletion/Substitution/Addition (DSA)	Theory	Sinisi and van der Laan, 2004 [32]	*dsa* package in R
	Application	Mortimer et al., 2008 [33]	
Least absolute shrinkage and selection operator (LASSO)	Theory	Tibshirani, 1996 [53];	*lars* package in R
		Efron et al., 2004 [54]	
	Application	Roberts and Martin, 2005 [42]	
Partial least-square regression (PLSR)	Theory	Hoeskuldsson, 1988 [40]	*pls* package in R
	Application	N/A	
Supervised principal component analysis (SPCA)	Theory	Bair et al., 2006 [52]	N/A
	Application	Roberts and Martin, 2006 [38]	
Classification and regression tree (CART)	Theory	Breiman et al., 1984 [48]	*rpart* package in R
	Application	Hu et al., 2008 [31]	

From our empirical investigation, we observe that in general, LASSO regression is an appealing approach due to its robustness in estimation of regression coefficients and its power in identifying non-zero coefficients associated with variables of importance under various study designs and parameter settings. By jointly minimizing the sum of squared errors and shrinking some estimated regression coefficients to zero, it seeks a model with accurate estimates in terms of mean squared error properties, especially when the exposure-response association is weak or multiple significant pollutants exist, as illustrated in the simulation studies and examples. Another feature of LASSO regression worth consideration is its computational efficiency. Given a long list of exposure variables, the LAR algorithm enables the implementation of LASSO regression to be on the same order of the ordinary least squares, which is orders of magnitude more efficient than other methods such as DSA [54]. However, when there is a large pool of correlated candidate variables, LASSO regression tends to select conservative models by including unrelated predictors and impose a strong shrinkage towards zero on estimated regression coefficients.

As a competitive approach addressing the issue of model uncertainty by averaging across models, we find BMA useful in model selection and parameter estimation when the dataset has a high signal-to-noise ratio or a sufficiently large sample size, for instance, simulated datasets in Scenarios 3 and 4. When applied to realistic dataset in which the coefficient of determination *R*^*2*^ is small, BMA is likely to produce biased and inefficient estimates with both sampling and misspecification errors considered, as a result, true effects could be masked [45]. Another difficulty in BMA analysis is the selection of prior specifications on individual parameters and model space [57]. In the simulation studies and data analyses, we chose standard non-informative priors for all parameters and assumed all models to be equally likely. However, enumeration of all models causes computational inefficiency when exploring model spaces with more than 30 variables [63]. Therefore, it is encouraged to use informative priors or reduced set of models to improve the predictive accuracy if background knowledge is possible [72]. Furthermore, the idea of averaging over models using different shrinkage weights, approximate the corresponding marginal probabilities by BIC for faster computation are aspects of BMA analysis that deserve more attention.

PCA-based methods, such as SPCA and PLSR, may reduce the impact of multicollinearity, but the bias in their effect estimates is considerable. This is not surprising because there is no guarantee that maximum-variance predictor variables always maximize information from the dependent variable [73]. In our analysis, estimation for interactions was more consistent than for the main effects in PCA-based methods, and this may relate to the larger variability of the interaction terms which leads to larger loading factors in the construction of first few principal components and smaller biases in the estimated effects. PLSR in general has better estimation performance, especially for main effects, because all candidate predictors are considered in the model, while in SPCA individual exposures with non-zero main effects could be eliminated at the pre-screen because their univariate associations with the outcome are weak in the presence of non-zero interaction effects. When the property of feature selection is considered, SPCA appears to perform well given its ability of capturing non-zero coefficients in a relatively small model.

It is challenging to detect interaction effects among multiple pollutants using standard/generalized linear regression, so it is expected the DSA algorithm proposed for genomic data could be borrowed to address this problem. Unfortunately, from our analysis, DSA is not successful in identifying interactions either in the NHANES example or in simulated datasets, where the coefficient of determination *R*^*2*^ is low. It is possible that when applied to datasets with strong exposure-response associations or sufficiently large sample sizes, DSA would be helpful in identifying interactions or producing unbiased effect estimates.

From our analysis, LASSO regression is powerful at capturing non-zero coefficients. However, proper significance testing and inference following LASSO to produce honest p-values is a non-trivial task. Fitting an ordinary GLM with selected predictors and using the corresponding tests and confidence intervals are obviously incorrect and greedy solutions. Recent research in this domain has the potential to create breakthroughs in statistical inference following model selection [74]. To address the asymptotic problem in coefficient estimation, the adaptive LASSO regression has been proposed by introducing adaptive weights to the L_1_ penalty term [55]. Suppose $\hat{\beta }$ is a$\;\sqrt{n}$ -consistent estimate to *β *(e.g., $\hat{\beta }$ estimated by OLS) and *w* is a weights vector $w=1/|\hat{\beta }{|}^{\gamma }$ for some positive constant *γ*, the adaptive LASSO estimates are given by:

(11) $${\hat{\beta }}_{\mathrm{adaptiveLASSO}}=\arg\;\underset{\beta }{\text{min}}(||Y-\mathrm{Z\beta}|{|}_{2}^{2}+\lambda \sum_{p=1}^{P}w_{p}|\beta_{p}|)$$

It has been shown that the adaptive LASSO can enjoy the computational efficiency by performing the LARS algorithm on weighted predictors and achieve both consistency in variable selection and asymptotic normality in coefficient estimation [55]. To control the bias in effect estimation when predictors are highly correlated, an extension of LASSO regression, the group LASSO, could be used [75,76]. The group LASSO regression has a penalty as an intermediate between the *L*_*1*_ and *L*_*2*_ penalty with its estimator defined as:

(12) $${\hat{\beta }}_{\mathrm{groupLASSO}}=\arg\;\underset{\beta }{\text{min}}(||Y-\mathrm{Z\beta}|{|}_{2}^{2}+\lambda \sum_{g=1}^{G}\left||V_{g}\beta^{\left(g\right)}|{|}_{2}\right)$$

In this equation, $||Y-\mathrm{Z\beta}|{|}_{2}^{2}=\sum_{i=1}^{N}{\left(Y_{i}-\beta_{0}-\sum_{p=1}^{P}\beta_{p}Z_{\mathrm{ip}}\right)}^{2}$, *λ* ≥ 0 is a tuning parameter, $V_{g}=\sqrt{p_{g}}I$ is a penalty matrix with the number of predictors in the *g*-*th* group *p*_*g*_ and identity matrix *I*, and *β*^(g)^ is the coefficient vector of the *g-th* group, *g* = 1,…,*G*. It has the attractive property of performing variable selection at the group level and is invariant under groupwise orthogonal transformations like ridge regression [75,77]. Another alternative that we have not explored is the application of the elastic net, a penalized least squares method using a penalty as the a combination of the LASSO and ridge penalty [43,78]

(13) $${\hat{\beta }}_{\mathrm{elastic}-\mathrm{net}}=\arg\;\underset{\beta }{\text{min}}\left(||Y-\mathrm{Z\beta}|{|}_{2}^{2}+\lambda_{2}||\beta |{|}_{2}^{2}+\lambda_{1}||\beta |{|}_{1}\right)$$

where $||\beta |{|}_{2}^{2}=\sum_{p=1}^{P}\beta_{p}^{2}$, $||\beta |{|}_{1}=\sum_{p=1}^{P}\left|\beta_{p}\right|$, λ_*1*_ and *λ*_*2*_ refer to the tuning parameters for *L*_*1*_ and *L*_*2*_ norm penalty. As a compromise between ridge and lasso regression, the elastic net has the characteristic of both selecting variables like LASSO and shrinking the regression coefficients of correlated predictors like ridge. Therefore, it outperforms LASSO regression by encouraging grouping effects and improving the prediction accuracy when there are high correlations between predictors [43,78]. Both the group LASSO regression and the elastic net are popular choices when dealing with grouped variables, a data structure matches well with the hierarchical nature of the pollution mixture, and hence deserves further investigation.

As a nonparametric technique, CART is well suited for exploring complex relationships under a multipollutant framework, however, it is limited by the fact that enforcing monotonicity constrains in building a regression tree is not possible. Some ideas have been provided in the construction of monotone classification trees. Potharst and Bioch suggested imposing monotonicity constraints by adding corner elements of nodes to the existing data [79]. Feelders proposed to use resampling to generate many different trees and select the ones that are monotone [80]. Feelders and Pardoel have developed an algorithm that grows a large overfitted tree at an initial step, prunes the tree towards a sequence of monotone subtrees, and then selects the one with the best predictive accuracy [81]. Hopefully, some of these algorithms can be improved and extended to the monotone regression trees, making the CART more attractive in describing exposure-response relationships.

In this study, we proposed a two-step strategy for estimating health effects when a large number of candidate pollutants exist. As an initial step, we use CART to explore the associations between individual pollutants and the response. At the second step, different methods (BMA, DSA, LASSO, PLSR, and SPCA) are applied to the subset of important pollutants selected by CART. The advantage of this two-step strategy comes from the reduction of some less important dimensions at the first step, and consequently the signal in the dataset carried forward to the second step is boosted. However, in real data analysis, there is possibility that a true effect failed to be identified at the initial step given a weak exposure-response association will be missed in the regression model. Therefore, the performance of this two-step strategy depends heavily on the initial screen by CART. Compared to univariate associations assessed in the first step of SPCA, CART uses the information from the set of all candidate variables as a whole and examines the complex interactions among exposures and responses, thus we expect this two-step modeling strategy to be adaptable in a multipollutant context.

One limitation of our study is that only linear main effects and linear interaction terms between pollutants were assumed in our simulation and data analyses, however, this assumption may not always be true as evidences of nonlinear relationship, such as threshold effect, polynomial effect or other non-parametric relationship, have been provided in multiple studies [10,82-84]. Among all the methods we have discussed, LASSO regression and BMA perform well in the current simulation studies but may cause bias in estimation if a misspecified interaction model was adopted in the presence of a truly nonlinear effect; DSA algorithm exhibits limited ability in detecting interactions in our simulations but may perform better in higher order polynomial models; CART may help to identify the threshold values of exposure variables and capture non-linear and higher order interactions but does not quantify exposure effect estimates. More expanded simulation studies with complex and high-dimensional non-linear exposure-response surface are warranted. We also acknowledge that although we have examined multiple simulation scenarios in our study, the estimated health effects of exposures in some time‒series studies are much smaller than the effects defined in our simulations [4,7,10], thus further assessment of the relative performances of different methods in time-series analysis with smaller effect size or larger sample size may be of added value. Another major issue in exposure epidemiology, namely, exposure measurement error and varying limits of detection for different exposures was not considered in our study, it has become well recognized that measurement errors at different scales may bias the estimation of regression coefficients in a multipolluant model, for instance, effects of pollutants measured with larger errors can be transferred to other correlated pollutants with less errors [85]. Researchers have taken steps to address the challenges of measurement error in time-series analyses [85-87], and further exploration is needed to develop methods that take into account the exposure uncertainty. Selection of lags in exposure-response relationship is another important issue we did not explore. How to incorporate multiple lagged exposures in a high-dimensional response-exposure surface remains a problem where no consensus has been reached. Selecting predictors/complex models under a distributed lag structure remains an issue of ongoing research [88,89]. In many health effects studies, where direct measurements of personal exposure to multiple pollutants are not practical, ambient pollutants concentrations are often used as proxies for personal exposure [6,13]. However, previous studies suggested that exposure assessment based upon regional monitors does not adequately represent the personal exposure [1,90-92]. Approaches to estimating personal exposure levels from ambient measurements via spatial modeling has been adopted for some air pollutants (e.g. NO_2_, PM_2.5_) [9,93,94], but no attempt towards creating a multipollutant spatially varying surface has been reported. Use of surrogates as imperfect measures of environmental exposures or imputation strategies to circumvent incomplete data issues is not studied in this paper.

## Conclusions

Among the five methods evaluated for regression analysis, there is no uniform dominance of one method across all examined simulation scenarios and data examples. Assuming that exposure distributions are reasonably approximated by lognormal distributions and the strength of correlations among pollutants is moderate, the performances of competing methods differ according to the nature of the response variable, the sample size, the number of exposure variables involved, and the strength of exposure-response association. In addition, supported by our results, the two-step modeling strategy proposed in this paper is potentially applicable under a multipollutant framework by taking advantage of both the screening feature of CART and dimension reduction or variable selection property of the subsequent statistical method. Extension of these methods under complex sampling schemes and correlated data with accompanying software packages merits further development.

Characterizing uncertainty in appropriate ways to proceed with tests of significance and construction of valid confidence intervals (often with biased estimates) following the variable selection/shrinkage methods we have discussed is a very important problem that is not fully resolved. This is typically done by advanced bootstrap or resampling strategies that is non-trivial and computation intensive. Bayesian methods have an advantage of providing measures of uncertainty based on the exact draws from a posterior distribution, an idea that translates to complex models. In this paper we have not discussed fully Bayesian methods. Bayesian approaches require a complete and thorough independent study.

Modeling of non-linear and complex exposure surfaces that are truly high dimensional is a daunting problem, recent attempts toward targeted estimation of parameters that are relevant for policy-making using reduced hierarchical models are extremely noteworthy in this context [95]. After building a complex model, one may attempt to extract information on not just the statistical model parameters, but parameters that are most informative regarding the scientific or policy question of interest. For example, risk ratios corresponding to days when certain pollutants exceed national standards may be more interpretable than the parameters associated with a natural spline term in a non-linear model [95]. However, any model trying to capture simultaneous co-exposure due to multiple pollutants comes with the challenge of sparsity of sample size in cross-configuration levels of pollutants, thus borrowing strength from prior studies, meta-analysis across studies and the possibility of using variable selection shrinkage methods, smoothing techniques as well as Bayesian hierarchical models offer modern statistical tools to approach this problem. The ultimate goal will be to arrive at a low-dimensional, meaningful representation or summary of a high dimensional modeling problem.

## Abbreviations

PM2.5: Particulate matter less than 2.5 micrometers in diameter; PM10-2.5: Particulate matter between 2.5 and 10 micrometers in diameter; CO: Carbon monoxide; SO2: Sulfur dioxide; NO2: Nitrogen dioxide; O3: Ozone; BMA: Bayesian model averaging; DSA: Deletion/Substitution/Addition; LASSO: Least absolute shrinkage and selection operator; PLSR: Partial least-square regression; SPCA: Supervised principal component analysis; PACF: Partial autocorrelation function; DOW: Day of the week; Temp: Temperature; RH: Relative humidity; NHANES: National Health and Nutrition Examination Survey; DAMAT: Detroit Asthma Morbidity, Air Quality and Traffic; FPR: False positive rate; TPR: True positive rate; MSE: Mean squared error.

## Competing interests

The authors declared that they have no competing interests.

## Authors’ contributions

ZS conducted simulation studies, performed data analysis of the second example, and drafted the manuscript. YT assisted in simulation studies, performed statistical analysis of the first example, and drafted portions of the article. SL has been involved in data cleaning and statistical analysis of the second example, and drafted portions of the article. KKF helped to clean data for the first example, drafted portions of the article, and edited the article carefully. JDM designed the study of the first example and edited the article. SKP provided insightful comments. SAB designed the DAMAT study and revised the article critically. BM conceived the study, directed its implementation and coordination, provided statistical methodology insights, and edited the article. All authors have read and approved the final manuscript.

## Supplementary Material

Additional file 1 Annotated R-codes. Click here for file

Additional file 2 Supplemental material. Click here for file
