# Supplementary material for: Statistical strategies for constructing health risk models with multiple pollutants and their interactions: possible choices and comparisons
Source: Environ Health. 2013 Oct 4;12:85. doi: 10.1186/1476-069X-12-85 (PMC3857674; doi:10.1186/1476-069X-12-85)
Supplement: Additional file 1 — Annotated R-codes. [file 1476-069X-12-85-S1.docx]

**Appendix: annotated R-codes**

library(mvtnorm) #generate multivariate normal

library(compositions) #generate multivariate log-normal

library(DSA) #DSA algorithm

library(superpc) #supervised PCA - cross-sectional

library(rpart) #Regression Tree

library(lars) #lasso regression

library(glmnet) #elastic net

library(pls) #partial least square regression

library(plsRglm) #partial least square regression for GLM

library(BMA) #Bayesian Model Averaging

library(splines) #natural spline function

library(mgcv) #generalized additive model (GAM)

#################### Data generation for Scenarios 1: ######################

################# Cross-sectional study with 4 pollutants ##################

## Parameters for exposure variables in a multivariate way

# mean and covariance matrix of exposure variables

meanExpo = c(1.20, 2.30, 1.89, 1.00)

varExpo = matrix(c(1, 0.52, 0.35, 0.28,

0.52, 1, 0.57, 0.54,

0.35, 0.57, 1, 0.41,

0.28, 0.54, 0.41, 1), nrow=4, byrow=T)

# mean and covariance matrix of the log of exposure variables

sigma.tilde = matrix(0, nrow=4, ncol=4)

for(i in 1:4) {

for(j in 1:4) {

sigma.tilde[i,j] = log(1+(varExpo[i,j]/(meanExpo[i]*meanExpo[j])))

}

}

mu.tilde = rep(0,4)

for(i in 1:4) {

mu.tilde[i] = log(meanExpo[i])-sigma.tilde[i,i]/2

}

###### Simulation results from 1,000 iterations

n=1000

N=250

# generate empty vectors or matrices for storing simulation results

b2.dsa = c(); b3.dsa = c(); g23.dsa = c(); size.dsa = c();

pc1.b2.spca = c(); pc1.b3.spca = c(); pc1.g23.spca = c(); size.spca = c();

b2.lasso = c(); b3.lasso = c(); g23.lasso = c(); size.lasso = c()

b2.lcr = c(); b3.lcr = c(); g23.lcr = c(); size.lcr = c(); se.b2.lcr = c(); se.b3.lcr = c(); se.g23.lcr = c();

beta.mat.plsr = matrix(nrow=n, ncol=10);

predictor.cart = c(); node.cart = c();

prob.bma = c(); beta.bma = c(); se.bma = c();

for (iter in 1: n) {

# Generate 4 exposure variables

simExpo = rlnorm.rplus(N, mu.tilde, sigma.tilde)

X1_s = simExpo[,1]

X2_s = simExpo[,2]

X3_s = simExpo[,3]

X4_s = simExpo[,4]

# Generate outccome Y from a linear regression model

error = rnorm(N, 0, 1)

Y = 0.1 + 0.5*X2_s + 0.5*X3_s + 0.2*X2_s*X3_s + 3*error

scenario1 = cbind(Y, X1_s, X2_s, X3_s, X4_s)

colnames(scenario1) = c("Y", "X1", "X2", "X3", "X4")

# Predictor matrix (p by n matrix, one observation (p features) per column)

X1_X2 = X1_s*X2_s

X1_X3 = X1_s*X3_s

X1_X4 = X1_s*X4_s

X2_X3 = X2_s*X3_s

X2_X4 = X2_s*X4_s

X3_X4 = X3_s*X4_s

predictors.scenario1 = t(as.matrix(cbind(X1_s, X2_s, X3_s, X4_s, X1_X2, X1_X3, X1_X4, X2_X3, X2_X4, X3_X4))) #transpose

featurenames.scenario1 = c("X1", "X2", "X3", "X4", "X1*X2", "X1*X3", "X1*X4", "X2*X3", "X2*X4,

"X3*X4")

rownames(predictors.scenario1) = featurenames.scenario1

######## Method I. Deletion/Substitution/Addition ########

# apply DSA algorithm

scenario1.dsa = DSA(Y ~ X1 + X2 + X3 + X4, data = as.data.frame(scenario1), maxsize = 8,

maxsumofpow = 2, maxorderint = 2, nsplits=10)

var.temp = c(colnames(scenario1.dsa$coefficients)[1], colnames(scenario1.dsa$coefficients)[2],

colnames(scenario1.dsa$coefficients)[3], colnames(scenario1.dsa$coefficients)[4],

colnames(scenario1.dsa$coefficients)[5], colnames(scenario1.dsa$coefficients)[6],

colnames(scenario1.dsa$coefficients)[7], colnames(scenario1.dsa$coefficients)[8])

var.dsa = cbind(var.dsa, var.temp)

# number of terms selected in the DSA model

size.dsa = c(size.dsa, max(which(!is.na(var.temp))))

# match function returns the location of exposure variables

b2.dsa = c(b2.dsa, scenario1.dsa$coefficients[match("I(X2^1)", var.temp)])

b3.dsa = c(b3.dsa, scenario1.dsa$coefficients[match("I(X3^1)", var.temp)])

g23.dsa = c(g23.dsa, scenario1.dsa$coefficients[match("I(X2^1 * X3^1)", var.temp)])

######## Method II. Supervised PCA (SPCA) ########

# create train and test data objects, here we use the same data set.

data.spca = list(x=predictors.scenario1,y=Y, featurenames=featurenames.scenario1)

# train the model: computes Z score for each predictor

train.obj = superpc.train(data.spca, type="regression")

# cross-validate the model

cv.obj = superpc.cv(train.obj, data.spca, min.features=0)

# choose the best threshold theta

thresholds.spca = cv.obj$scor[1,][!is.na(cv.obj$scor[1,])]

k.spca = cv.obj$thresholds[which(thresholds.spca == max(thresholds.spca))]

# derive reduced matrix Z’ for model fitting

fit.cts = superpc.predict(train.obj, data.spca, data.spca, threshold=k.spca-1, n.components=1, prediction.type="continuous")

red.predictors = mat.or.vec(10, N)

for (i in 1:10){

red.predictors[i,] = fit.cts$which.features[i]*predictors.scenario1[i,]

}

rownames(red.predictors) = featurenames.scenario1

red.predictors =red.predictors[which(rowSums(red.predictors)>0),] #remove rows of 0s

red.features = featurenames.scenario1[fit.cts$which.features == T]

s.spca = sum(fit.cts$which.features) # number of terms after selection

size.spca = c(size.spca, s.spca)

centered.spca = red.predictors - rowMeans(red.predictors)

cov.spca = centered.spca %*% t(centered.spca) / (N-1)

# Fucntion: compute first several PCs of the reduced matrix Z’

princomp = function(pc) {

pred.red.predictors = t(svd(cov.spca)$u[,1:pc]) %*% red.predictors

scenario1.spca = lm(Y ~ t(pred.red.predictors))

lm.b = c()

for(j in 1:pc){ # extract coeff from linear model for PCs

lm.b = c(lm.b, scenario1.spca$coef[[j+1]])

}

beta = real(s)

for(j in 1:s){ # compute betas in the final model

beta[j] = sum(svd(cov.spca)$u[j,1:pc]*lm.b)

}

return(beta)

}

# Apply function and extract coefficients from linear model

fun1 = princomp(1)

pc1.b2.spca = c(pc1.b2.spca, fun1[red.features == "X2"])

pc1.b3.spca = c(pc1.b3.spca, fun1[red.features == "X3"])

pc1.g23.spca = c(pc1.g23.spca, fun1[red.features == "X2*X3"])

######## Method III. lasso regression ########

# Covariate matrix: add 1st column as intercept

Z.lasso = cbind(1, t(predictors.scenario1)) #add 1st column as intercept

# LASSO for main effects + interactions

scenario1.lasso = lars(Z.lasso, Y, type="lasso")

betas.lasso = scenario1.lasso$beta[match(min(scenario1.lasso$Cp), scenario1.lasso$Cp),]

# extract LASSO coefficients

b2.lasso = c(b2.lasso, betas[[3]]) #X2

b3.lasso = c(b3.lasso, betas[[4]]) #X3

g23.lasso = c(g23.lasso, betas[[9]]) #X2*X3

# model size (intercept not included)

if (betas.lasso[[1]] == 0.00000000)

{s.lasso = sum(betas.lasso != 0.00000000)}

else {s.lasso = sum(betas.lasso != 0.00000000)-1}

size.lasso = c(size.lasso, s.lasso)

######## Method IV. Partial least square regression (PLSR) ########

# Covariate matrix: transposed

Z.plsr = t(predictors.scenario1)

# Choose optimum no.of components by Root Mean Squared Error of Prediction in cross validation

opt.pcr = plsr(Y~Z.plsr, ncomp=10, validation="CV")

err.CV = c()

for (i in 1:10) {err.CV[i] = RMSEP(opt.pcr)$val[i*2+1]}

delta = err.CV[1:9] - err.CV[2:10] # delta vector contains RMSEP differences

comp.plsr = min(which(delta<0.05))

# mixed model regression coefficients

scenario1.plsr = plsr(Y~Z.plsr, ncomp = comp.plsr)

# extract regression ocefficients

beta.mat.plsr[iter, ] = coef(scenario1.plsr, ncomp = comp.plsr, intercept = TRUE)[2:11]

######## Method V. Classification & regression tree ########

# grow tree

scenario1.cart = rpart(Y ~ X1 + X2 + X3 + X4, method = "anova", data = scenario1)

# prune the tree by min CP

scenario1.pcart = prune(scenario1.cart,

cp=scenario1.cart$cptable[which.min(scenario1.cart$cptable[, "xerror"]), "CP"])

# number of terminal nodes

k.cart = sum(scenario1.pcart$frame$var=="<leaf>")

node.cart = c(node.cart, k.cart)

# extract all unique variables included in CART

j = dim(scenario1.pcart$frame[1])[1]

var = c()

for (i in 1:j)

{ var = c(var, scenario1.pcart$frame[i,1]) }

predictor.cart = c(predictor.cart, unique(var[var!=1]-1))

######## Method VI. Bayesian Model Averaging (BMA) ########

# Covariate matrix: transposed

Z.bma = t(predictors.scenario1)

# apply BMA

scenario1.bma = bicreg(Z.bma, Y)

# extract coefficients

prob.bma = rbind(prob.bma, scenario1.bma$probne0[2:11])

beta.bma = rbind(beta.bma, scenario1.bma$postmean[2:11])

se.bma = rbind(se.bma, scenario1.bma$postsd[2:11])

}

## summary function 1 to compute Mean, empirical SE and percent in the model of each predictor

summary1 = function(x) {

# remove NA/0 elements

x = x[!is.na(x)]; x = x[x!=0];

list(mean=mean(x), emp.se=sd(x)/sqrt(length(x)), power=length(x)/n)

}

## summary function 2 to compute Mean, model-based SE, empirical SE and percent in the model of ## each predictor

summary2 = function(x, y) {

x = x[!is.na(x)]; x = x[x!=0];

y = y[!is.na(y)]; y = y[y!=0];

list(mean=mean(x), mod.se=mean(y), emp.se=sd(x)/sqrt(length(x)), power=length(x)/n)

}

#### Summary results from DSA model

# average size

mean(size.dsa)-1

# Mean, empirical SE and percent

summary1(b2.dsa)

summary1(b3.dsa)

summary1(g23.dsa)

#### Summary results from SPCA model

# average size

mean(size.spca)

# Mean, empirical SE and percent

summary1(pc1.b2.spca)

summary1(pc1.b3.spca)

summary1(pc1.g23.spca)

#### Summary results from LASSO model

# average size

mean(size.lasso)

# Mean, empirical SE and percent

summary1(b2.lasso)

summary1(b3.lasso)

summary1(g23.lasso)

#### Summary results from PLSR model

colnames(beta.mat.plsr) = featurenames.scenario1

colMeans(beta.mat.plsr)

#### Summary results from CART model

# average size of the tree (number of terminal nodes)

mean(node.cart)

table(node)

# percent of exposure retain in the model

sum(predictor.cart == 1)*100/n

sum(predictor.cart == 2)*100/n

sum(predictor.cart == 3)*100/n

sum(predictor.cart == 4)*100/n

#### Summary results from BMA model

# mean, model-based SE and percent of coefficients

colnames(prob.bma) = colnames(beta.bma) = colnames(se.bma) = featurenames.scenario1

table.bma = cbind(colMeans(beta.bma), colMeans(se.bma), colMeans(prob.bma))

colnames(table.bma) = c("beta", "se", "P")

table.bma

# average size

sum(prob.bma/100)/n

#################### Data generation for Scenarios 2: ######################

################# Cross-sectional study with 20 pollutants #################

## Parameters for exposure variables (X matrix) in a multivariate way

## Assume x1, x2,..., x20 follow lognormal distributions with standardized mean (SD): 1(1)

# mean and covariance matrix of exposure variables

meanExpo = rep(1, 20)

Cov1 = matrix(c( 1, 0.2, 0.2, 0.2, 0.2,

0.2, 1, 0.2, 0.2, 0.2,

0.2, 0.2, 1, 0.2, 0.2,

0.2, 0.2, 0.2, 1, 0.2,

0.2, 0.2, 0.2, 0.2, 1), nrow=5, byrow=T)

Cov2 = matrix(rep(0,75), nrow=5, byrow=T)

Cov3 = matrix(rep(0,75), nrow=15, byrow=T)

Cov4 = diag(rep(1,15))

varExpo = rbind(cbind(Cov1, Cov2), cbind(Cov3, Cov4))

# mean and covariance matrix of the log of exposure variables

sigma.tilde = matrix(0, nrow=20, ncol=20)

for(i in 1:20) {

for(j in 1:20) {

sigma.tilde[i,j] = log(1+(varExpo[i,j]/(meanExpo[i]*meanExpo[j])))

}

}

mu.tilde = rep(0,20)

for(i in 1:20) {

mu.tilde[i] = log(meanExpo[i])-sigma.tilde[i,i]/2

}

# Assign names to predictors (pollutants and interactions)

pollutants = c()

for (i in 1:20) { pollutants [i] = paste("X", i, sep = "") }

int2 = c()

for (i in 1:19) {

for (j in (i+1):20) {

int2 = c(int2, paste("X", i, "_", j, sep = ""))

}

}

###### Simulation results from 1,000 iterations

n=1000

N=250

# generate empty data frames or matrices for storing simulation results

beta.dsa = data.frame(); name.dsa = data.frame();

pc1.beta.spca = matrix(nrow=n, ncol=210); size.spca = c();

beta.mat.lasso = matrix(nrow=n, ncol=210); size.lasso = c()

beta.mat.plsr = matrix(nrow=n, ncol=210)

predictor.cart = c(); node.cart = c();

b1.bma=c(); b2.bma=c(); b6.bma=c(); b7.bma=c(); g12.bma=c(); g16.bma=c(); g67.bma=c(); size.bma=c();

for (iter in 1: n) {

# Generate 20 exposure variables

simExpo = rlnorm.rplus(N, mu.tilde, sigma.tilde)

for (i in 1:20) {assign(paste("X", i, sep = ""), simExpo[,i])}

# Generate outccome Y from a linear regression model, Y|predictors

error = rnorm(N, 0, 1)

Y = 0.1+0.5*X1+0.5*X2+0.5*X6+0.5*X7+0.2*X1*X2+0.2*X1*X6+0.2*X6*X7+3*error

scenario2 = cbind(Y, simExpo)

colnames(scenario2) = c("Y", pollutants)

# Predictor matrix

predictors.scenario2 = matrix(nrow=210, ncol=N)

predictors.scenario2[1:20,] = t(simExpo)

a=21

for (i in 1:19) {

for (j in (i+1):20) {

predictors.scenario2[a,] = simExpo[,i]*simExpo[,j];

a = a+1;

}

}

featurenames.scenario2 = c(pollutants, int2)

rownames(predictors.scenario2) = featurenames.scenario2

######## Method I. Deletion/Substitution/Addition (DSA) ########

# apply DSA algorithm

scenario2.dsa = DSA(Y ~ X1+X2+X3+X4+X5+X6+X7+X8+X9+X10+X11+X12+X13+X14+X15+X16+X17+X18+X19+X20, data = as.data.frame(scenario2), maxsize = 30, maxsumofpow = 2, maxorderint = 2, nsplits=10)

p.dsa = length(colnames(scenario2.dsa$coefficients))

for (i in 1:p.dsa) {

name.dsa[iter, i] = colnames(scenario2.dsa$coefficients)[i];

beta.dsa[iter, i] = scenario2.dsa$coefficients[i];

}

######## Method II. Supervised PCA (SPCA) ########

# create train and test data objects, here we use the same data set

data.spca = list(x=predictors.scenario2, y=Y, featurenames=featurenames.scenario2)

# train the model: compute Z score for each predictor

train.obj = superpc.train(data.spca, type="regression")

# cross-validate the model

cv.obj = superpc.cv(train.obj, data.spca, min.features=0)

# choose the best threshold theta

thresholds.spca = cv.obj$scor[1,][!is.na(cv.obj$scor[1,])]

k.spca = cv.obj$thresholds[which(thresholds.spca == max(thresholds.spca))]

# derive reduced matrix Z’ for model fitting

fit.cts = superpc.predict(train.obj, data.spca, data.spca, threshold=k.spca-1, n.components=1, prediction.type="continuous")

red.predictors = matrix(nrow=210, ncol=N)

for (i in 1:210){

red.predictors[i,] = fit.cts$which.features[i]* predictors.scenario2[i,]

}

rownames(red.predictors) = featurenames.scenario2

red.predictors = red.predictors[which(rowSums(red.predictors) > 0),] #remove rows of 0s

red.features = featurenames.scenario2[fit.cts$which.features == T]

s.spca = length(red.features) # number of terms after selection

size.spca = c(size.spca, s.spca)

centered.spca = red.predictors - rowMeans(red.predictors)

cov.spca = centered.spca %*% t(centered.spca) / (N-1)

# Fucntion: compute 1st PC of the reduced matrix Z’, PC can be extended to 3

princomp = function(pc) {

pred.red.predictors = t(svd(cov.spca)$u[,1:pc]) %*% red.predictors

scenario2.spca = lm(Y ~ t(pred.red.predictors))

lm.b = c()

for(j in 1:pc){ # extract coeff from linear model for PCs

lm.b = c(lm.b, scenario2.spca$coef[[j+1]])

}

beta = real(s)

for(j in 1:s){ # compute betas in the final model

beta[j] = sum(svd(cov.spca)$u[j,1:pc]*lm.b)

}

return(beta)

}

# Apply function and extract coefficients from linear model

fun1 = princomp(1)

for (i in 1:20) {

if (sum(red.features == paste("X", i, sep=""))>0) {

pc1.beta.spca[iter, i] = fun1[red.features == paste("X", i, sep="")];

}

else { pc1.beta.spca[iter, i] = NA; }

}

a=21

for (i in 1:19) {

for (j in (i+1):20) {

if (sum(red.features == paste("X", i, "_", j, sep=""))>0) {

pc1.beta.spca[iter, a] = fun1[red.features == paste("X", i, "_", j, sep="")];

}

else { pc1.beta.spca[iter, a] = NA; }

a=a+1

}

}

######## Method III. lasso regression ########

# Covariate matrix: add 1st column as intercept

Z.lasso = cbind(1, t(predictors.scenario2)) #add 1st column as intercept

# LASSO for main effects + interactions

scenario2.lasso = lars(Z.lasso, Y, type="lasso")

betas.lasso = scenario2.lasso$beta[match(min(scenario2.lasso$Cp), scenario2.lasso$Cp),]

# extract LASSO coefficients

for (j in 1:210) { beta.mat.lasso[iter,j] = betas.lasso[[j+1]] }

# model size (intercept not included)

if (betas.lasso[[1]] == 0.00000000) { s.lasso = sum(betas.lasso != 0.00000000) }

else { s.lasso = sum(betas.lasso != 0.00000000)-1 }

size.lasso = c(size.lasso, s.lasso)

######## Method IV. Partial least square regression (PLSR) ########

# Covariate matrix: transposed

Z.plsr = t(predictors.scenario2)

# Choose the optimum number of components by root mean squared error of prediction (RMSEP)

# in cross validation

opt.pcr = plsr(Y~Z.plsr, ncomp=210, validation="CV") # ncomp=210 for up to pairwise

err.CV = c()

for (i in 1:30) {err.CV[i] = RMSEP(opt.pcr)$val[i*2+1]}

delta = err.CV[1:29] - err.CV[2:30] # delta vector contains RMSEP differences

comp.plsr = min(which(delta<0.01))

# mixed model regression coefficients

scenario2.plsr = plsr(Y~Z.plsr, ncomp = comp.plsr)

# extract regression ocefficients

beta.mat.plsr[iter, ] = coef(scenario2.plsr, ncomp = comp.plsr, intercept = TRUE)[3:212]

######## Method V. Classification & regression tree ########

# grow the tree

scenario2.cart = rpart(

Y ~ X1+X2+X3+X4+X5+X6+X7+X8+X9+X10+X11+X12+X13+X14+X15+X16+X17+X18+X19+X20, method="anova", control=rpart.control(minsplit=n*0.01,cp=0.02),data=as.data.frame(scenario2))

# prune the tree by min CP

scenario2.pcart = prune(scenario2.cart, cp= scenario2.cart$cptable[which.min(scenario2.cart$cptable[, "xerror"]), "CP"])

# number of terminal nodes

k.cart = sum(scenario2.pcart$frame$var=="<leaf>")

node.cart = c(node.cart, k.cart)

# extract all unique variables included in CART

j = dim(scenario2.pcart$frame[1])[1]

var = c()

for (j in 1:j) { var = c(var, scenario2.pcart$frame[j,1]) }

predictor.cart = c(predictor.cart, unique(var[var!=1]-1))

######## Method VI. Bayesian Model Averaging (BMA) ########

# Covariate matrix: transposed

Z.bma = t(predictors.scenario2)

# apply BMA

scenario2.bma = bicreg(Z.bma, Y)

# extract coefficients

b1.bma=rbind(b1.bma, cbind(scenario2.bma$probne0[match("X1", scenario2.bma$namesx)],

scenario2.bma$postmean[match("X1", scenario2.bma$namesx)+1],

scenario2.bma$postsd[match("X1", scenario2.bma$namesx)+1]))

# +1 for intercept

b2.bma=rbind(b2.bma, cbind(scenario2.bma$probne0[match("X2", scenario2.bma$namesx)],

scenario2.bma$postmean[match("X2", scenario2.bma$namesx)+1],

scenario2.bma$postsd[match("X2", scenario2.bma$namesx)+1]))

b6.bma=rbind(b6.bma, cbind(scenario2.bma$probne0[match("X6", scenario2.bma$namesx)],

scenario2.bma$postmean[match("X6", scenario2.bma$namesx)+1],

scenario2.bma$postsd[match("X6", scenario2.bma$namesx)+1]))

b7.bma=rbind(b7.bma, cbind(scenario2.bma$probne0[match("X7", scenario2.bma$namesx)],

scenario2.bma$postmean[match("X7", scenario2.bma$namesx)+1],

scenario2.bma$postsd[match("X7", scenario2.bma$namesx)+1]))

g12.bma=rbind(g12.bma, cbind(scenario2.bma$probne0[match("X1_2", scenario2.bma$namesx)],

scenario2.bma$postmean[match("X1_2", scenario2.bma$namesx)+1],

scenario2.bma$postsd[match("X1_2", scenario2.bma$namesx)+1]))

g16.bma=rbind(g16.bma, cbind(scenario2.bma$probne0[match("X1_6", scenario2.bma$namesx)],

scenario2.bma$postmean[match("X1_6", scenario2.bma$namesx)+1],

scenario2.bma$postsd[match("X1_6", scenario2.bma$namesx)+1]))

g67.bma=rbind(g67.bma, cbind(scenario2.bma$probne0[match("X6_7", scenario2.bma$namesx)],

scenario2.bma$postmean[match("X6_7", scenario2.bma$namesx)+1],

scenario2.bma$postsd[match("X6_7", scenario2.bma$namesx)+1]))

# model size

size.bma = c(size.bma, sum(scenario2.bma$probne0)/100)

}

## Function to remove NA elements in the data frame;

NAremover = function(x) {

x = as.vector(x);

x = names[!is.na(x)];

return(x);

}

## Summary function 3 to compute Mean, empirical SE and percent in the model of each predictor

summary3 = function(x) {

x = x[!is.na(x)];

list(mean=mean(x), emp.se=sd(x)/sqrt(length(x)), power=length(x)/n)

}

#### Summary results from DSA model

# average size

length(NAremover(name.dsa))/n-1 # -1 for intercept

# beta coefficients

beta.dsa = NAremover(beta.dsa)

for (i in 1:20) {

assign(paste("b", i, sep=""), beta.dsa[which(names == paste("I(X", i, "^1)", sep="") )])

}

for (i in 1:19) {

for (j in (i+1):20) {

assign(paste("g", i, "_", j, sep=""),

beta.dsa[which(names == paste("I(X", i, "^1 * X", j, "^1)", sep="") )])

}

}

rbind(summary3(b1), summary3(b2), summary3(b6), summary3(b7),

summary3(g1_2), summary3(g1_6), summary3(g6_7))

#### Summary results from SPCA model

# average size

mean(size.spca)

# assign column names

colnames(pc1.beta.spca) = c(pollutants, int2)

# calculate percent of each predictor

percent.spca = c()

for (i in 1:210) {

x = pc1.beta.spca[,i]

percent.spca = c(percent.spca, length(x[!is.na(x)])*100/n)

}

# list names of predictors with a percentage > 20%

featurenames[which(percent.spca>20)]

# bate coefficients for predictors with a percentage > 20%

apply(pc1.beta.spca[,which(percent.spca > 20)], 2, summary3)

#### Summary results from lasso regression model

# average size

mean(size.lasso)

# calculate percent of each predictor

Percent.lasso = real(1000)

for (j in 1:210) {

percent.lasso[j] = sum(abs(beta.mat.lasso[,j]) > 0.00000000)/n

}

# bate coefficients

summary3(c(beta.mat.lasso[,match("X1", featurenames.scenario2)]))

summary3(c(beta.mat.lasso[,match("X2", featurenames.scenario2)]))

summary3(c(beta.mat.lasso[,match("X6", featurenames.scenario2)]))

summary3(c(beta.mat.lasso[,match("X7", featurenames.scenario2)]))

summary3(c(beta.mat.lasso[,match("X1_2", featurenames.scenario2)]))

summary3(c(beta.mat.lasso[,match("X1_6", featurenames.scenario2)]))

summary3(c(beta.mat.lasso[,match("X6_7", featurenames.scenario2)]))

#### Summary results from PLSR model

# beta coefficients for predictors “X1, X2, X6, X7, X1_2, X1_6, X6_7”

colnames(beta.mat.plsr) = featurenames.scenario2

colMeans(beta.mat.plsr[,c(1,2,6,7,21,25,106)])

#### Summary results from CART model

# average size of the tree (number of terminal nodes)

mean(node.cart)

table(node)

# percent of retain in the model

sum(predictor.cart==1)*100/n

sum(predictor.cart==2)*100/n

sum(predictor.cart==6)*100/n

sum(predictor.cart==7)*100/n

#### Summary results from BMA model

# average size

mean(size.bma)

table.bma = rbind(apply(b1.bma, 2, mean), apply(b2.bma, 2, mean), apply(b6.bma, 2, mean), apply(b7.bma, 2, mean), apply(g12.bma, 2, mean), apply(g16.bma, 2, mean), apply(g67.bma, 2, mean))

colnames(table.bma) = c("Percentage", "Beta", "SE")

rownames(table.bma) = c("X1", "X2", "X6", "X7", "X1_2", "X1_6", "X6_7")

table.bma

#################### Data generation for Scenarios 3: ######################

################### Time-series study with 4 pollutants ####################

# Calculate parameters of offsets from real data using GLM model

GAM1 = read.csv("Gam1.csv")

DAMAT = as.data.frame(cbind(GAM1$ER_Asthma, GAM1$Temp, GAM1$RH, GAM1$YEAR, GAM1$SEASON, GAM1$Weekday, GAM1$time))

colnames(DAMAT) = c("asthma", "temp", "RH", "year", "season", "DOW", "time")

DAMAT$time = c(1:366,1:365,1:365)

glm.1 = glm(asthma~factor(season)+factor(year)+factor(DOW)+ns(time,8)+ns(temp,4)+ns(RH,3), family=poisson, data=DAMAT)

offset = predict(glm.1) # offset on the log scale

# Partial autocorrelation function (PACF) up to lag10 from real data

pacf_X1 = c(0.3242, 0.0309, 0.0567, 0.0400, 0.0146, 0.0433, 0.0899, 0.0385, 0.0129, 0.0521)

pacf_X2 = c(0.5286, -0.0371, 0.0938, 0.0022, 0.0374, 0.1191, 0.0445, 0.0513, 0.0203, 0.0234)

pacf_X3 = c(0.8155, -0.3357, -0.0184, 0.0444, -0.0152, 0.0465, -0.0160, 0.0139, 0.0444, -0.0516)

pacf_X4 = c(0.3300, -0.0173, 0.0198, -0.0094, -0.0266, 0.0361, 0.0326, 0.0304, -0.0287, -0.0030)

pacf_matrix = cbind(pacf_X1, pacf_X2, pacf_X3, pacf_X4)

# compute the scales of the sum of PACF coefficients

scales = colSums(pacf_matrix)

# Seasonal effects

season1 = c(1.102197, 2.231360, 1.716842, 0.967068)

season2 = c(1.079931, 1.933108, 1.940799, 0.815423)

season3 = c(1.546554, 2.270622, 1.754159, 1.114600)

season4 = c(1.199649, 2.781179, 2.144554, 1.124027)

# Mean and covariance matrix of the 4 exposures

meanExpo = c(1.20, 2.30, 1.89, 1.00)

varExpo = matrix(c( 1, 0.52, 0.35, 0.28,

0.52, 1, 0.57, 0.54,

0.35, 0.57, 1, 0.41,

0.28, 0.54, 0.41, 1), nrow=4, byrow=T)

# mean and covariance matrix of the log of 4 exposures

sigma.tilde = matrix(0, nrow=4, ncol=4)

for(i in 1:4) {

for(j in 1:4) {

sigma.tilde[i,j] = log(1+(varExpo[i,j]/(meanExpo[i]*meanExpo[j])))

}

}

mu.tilde = rep(0,4)

for(i in 1:4) {

mu.tilde[i] = log(meanExpo[i])-sigma.tilde[i,i]/2

}

###### Simulation results from 1,000 iterations

n=1000

N=400

# generate empty vectors or matrices for storing simulation results

pc1.b1.spca = c(); pc1.b3.spca = c(); pc1.g13.spca = c(); size.spca = c();

b1.lasso = c(); b3.lasso = c(); g13.lasso = c(); size.lasso = c();

b1.lcr = c(); b3.lcr = c(); g13.lcr = c(); size.lcr = c(); se.b1.lcr = c(); se.b3.lcr = c(); se.g13.lcr = c();

beta.mat.plsr = matrix(nrow=n, ncol=10);

predictor.cart = c(); node.cart = c();

prob.bma = c(); beta.bma = c(); se.bma = c();

for (iter in 1: n) {

# Generate 4 exposures on the first ten days as starting points

Expo10 = rlnorm.rplus(10, mu.tilde, sigma.tilde)

simExpo = matrix(,600,4)

simExpo[1:10,] = Expo10

# Simulate 4 multivariate exposures by autoregressive models on previous 10 days AR(10)

for (t in 11:600){

temp = matrix(0,4,10)

for (i in 1:10){ temp[,i] = diag(pacf_matrix[i,]) %*% simExpo[t-i,] }

# include only positive values

sim = rep(0,4)

while(min(sim) <= 0){

if (t%%365<=92){

sim = rowSums(temp)+diag(1-scales)%*%season1+mvrnorm(1,rep(0,4), varExpo)

}

if (t%%365>92 && t%%365<=183){

sim = rowSums(temp)+diag(1-scales)%*%season2+mvrnorm(1,rep(0,4), varExpo)

}

if (t%%365>183 && t%%365<=275){

sim = rowSums(temp)+diag(1-scales)%*%season3+mvrnorm(1,rep(0,4), varExpo)

}

if (t%%365>275){

sim = rowSums(temp)+diag(1-scales)%*%season4+mvrnorm(1,rep(0,4), varExpo)

}

}

simExpo[t,] = sim

}

# Remove data after the burn-in phase

burnin = 200

simExpo = simExpo[(burnin+1):(burnin+400),]

# Distribute 4 exposures

X1 = simExpo[,1]

X2 = simExpo[,2]

X3 = simExpo[,3]

X4 = simExpo[,4]

lambda = exp(0.3*X1+0.3*X3+0.1*X1*X3)

# Rescale to keep the mean of mu the same scale of offset

mu = lambda*(exp(offset[1:400])/mean(lambda))

# Generate outcome Y by a two-stage Poission regression

Y = rep(0,400)

for (i in 1:400) { Y[i] = rpois(1, mu[i]) }

log_offset = offset[1:400]-log(mean(lambda))

# Predictor matrix

X1_X2 = X1*X2

X1_X3 = X1*X3

X1_X4 = X1*X4

X2_X3 = X2*X3

X2_X4 = X2*X4

X3_X4 = X3*X4

predictors.scenario3 = as.matrix(cbind(X1, X2, X3, X4, X1_X2, X1_X3, X1_X4, X2_X3, X2_X4, X3_X4))

featurenames.scenario3 = c("X1", "X2", "X3", "X4", "X1*X2", "X1*X3", "X1*X4", "X2*X3", "X2*X4", "X3*X4")

colnames(predictors.scenario3) = featurenames.scenario3

######## Method I. Deletion/Substitution/Addition ########

########## Fail to work under time-series study ########

######## Method II. Supervised PCA (SPCA) ########

#### Step 1-5 modified from SPCA by Dr. Steven Roberts ####

# step 1: Fit models containing a single pollutant using training data

wald = rep(0,10)

for (i in 1:10) {

glmfit = glm(Y ~ predictors.scenario3[,i], offset=log_offset, family=poisson);

wald[i] = (summary(glmfit)$coef[,3])[2]

}

# step 2: Sort the variables based on their importance (Wald statistics, large to small)

importance = rev(sort.list(abs(wald)))

# step 3: Find the principal components

# extract only first PC from 90% of the data as the training set

PC_training = matrix(nrow=360, ncol=10)

PC_training[,1] = predictors.scenario3[1:360, sort(importance[1])]

for (i in 2:10) {

PC_training[,i] = predictors.scenario3[1:360, sort(importance[1:i])] %*% prcomp(predictors.scenario3[1:360, sort(importance[1:i])])$rotation[,1]

}

# extract first PC from 10% of the data as the testing set

PC_testing = matrix(nrow=40, ncol=10)

PC_testing[,1] = predictors.scenario3[361:400, sort(importance[1])]

for (i in 2:10) {

PC_testing[,i] = predictors.scenario3[361:400, sort(importance[1:i])] %*% prcomp(predictors.scenario3[361:400, sort(importance[1:i])])$rotation[,1]

}

# step 4: Compute test errors

testerror = rep(0, 10)

for (i in 1:10) {

# fit models to the training set

PC = PC_training[,i]

log_mu = log_offset[1:360]

fit = glm(Y[1:360] ~ PC, offset=log_mu, family=poisson)

# find the perdiction error for each model using testing set

test = predict(fit, data.frame(PC=PC_testing[,i], log_mu=log_offset[361:400]), type="response")

testerror[i] = sum((test-Y[361:400])^2)

}

# step 5: The model with the lowest prediction error was then re-fitted to the entire dataset

# The number of covariates included into the "best" model

s.spca = which(testerror == min(testerror))

size.spca = c(size.spca, s.spca)

# list names of covariates included into the "best" model

f.spca = featurenames.scenario3[importance[1:s.spca]]

features.spca = c(features.spca, f.spca)

Z.spca = predictors.scenario3[, importance[1:s.spca]] # reduced covariates subset

# The loadings are estimated from the entire data and the model re-fitted

if (s.spca ==1) { PC_best = Z.spca }

if (s.spca >1) { PC_best = Z.spca %*% prcomp(Z.spca)$rotation[,1] }

# apply GLM model

scenario3.spca = glm(Y ~ PC_best, offset=log_offset, family=poisson)

beta.spca = scenario3.spca$coef[[2]] * prcomp(Z.spca)$rotation[,1]

if (s.spca ==1) { names(beta.spca)=featurenames.scenario3[importance[1]] }

# extract beta coefficients

pc1.b1.spca = c(pc1.b1.spca, beta.spca[names(beta.spca) == "X1"])

pc1.b3.spca = c(pc1.b3.spca, beta.spca[names(beta.spca) == "X3"])

pc1.g13.spca = c(pc1.g13.spca, beta.spca[names(beta.spca) == "X1*X3"])

######## Method III. lasso regression ########

# use glmnet package: alpha=1(lasso), 0(ridge), 0~1(elastic net)

scenario3.lasso = glmnet(predictors.scenario3, Y, family="poisson", offset=log_offset, alpha=1)

scenario3.lasso.cv = cv.glmnet(predictors.scenario3, Y, family="poisson", offset=log_offset, alpha=1)

betas.lasso = coef(scenario3.lasso, s=scenario3.lasso.cv$lambda.min)

# extract LASSO coefficients

b1.lasso = c(b1.lasso, betas.lasso [2])

b3.lasso = c(b3.lasso, betas.lasso [4])

g13.lasso = c(g13.lasso, betas.lasso [7])

# model size (intercept not included)

if (betas.lasso [1] == 0.00000000) { s.lasso = sum(betas.lasso != 0.00000000) }

else { s.lasso = sum(betas.lasso != 0.00000000)-1 }

size.lasso = cbind(size.lasso, s.lasso)

######## Method IV. Partial least square regression (PLSR) ########

data.plsr = data.frame(cbind(Y, predictors.scenario3, log_offset))

# Apply PLSR for glm function

scenario3.plsr = plsRglm(Y~predictors.scenario3, offset=log_offset, data=data.plsr, nt=10, model="pls-glm-poisson")

# use BIC to determine optimal number of components

comp.plsr = which(scenario3.plsr$InfCrit[,2] == min(scenario3.plsr$InfCrit[,2]))-1

# refit model and extract beta coefficients from the optimal model

opt.scenario3.plsr = plsRglm(Y~predictors.scenario3, offset=log_offset, data=data.plsr, nt=comp.plsr, model="pls-glm-poisson")

beta.mat.plsr[iter, ] = opt.scenario3.plsr$Coeffs[2:11]

######## Method V. Classification & regression tree ########

# grow the tree

scenario3.cart = rpart(Y ~ X1 + X2 + X3 + X4, method = "anova")

# prune the tree by min CP

scenario3.pcart = prune(scenario3.cart,

cp=scenario3.cart$cptable[which.min(scenario3.cart$cptable[, "xerror"]), "CP"])

# number of terminal nodes

k.cart = sum(scenario3.pcart$frame$var=="<leaf>")

node.cart = c(node.cart, k.cart)

# extract all unique variables included in CART

j = dim(scenario3.pcart$frame[1])[1]

var = c()

for (i in 1:j)

{ var = c(var, scenario3.pcart$frame[i,1]) }

predictor.cart = c(predictor.cart, unique(var[var!=1]-1))

######## Method VI. Bayesian Model Averaging (BMA) ########

# apply BMA

scenario3.bma = bic.glm(Y~offset(log_offset)+X1+X2+X3+X4+X1_X2+X1_X3+X1_X4+X2_X3+X2_X4+X3_X4, glm.family=poisson(), factor.type=F)

# extract coefficients

prob.bma = rbind(prob.bma, summary(scenario3.bma)[2:11,1])

beta.bma = rbind(beta.bma, summary(scenario3.bma)[2:11,2])

se.bma = rbind(se.bma, summary(scenario3.bma)[2:11,3])

}

#### Summary results from SPCA model

# average size

mean(size.spca)

# Mean, empirical SE and percent

summary1(pc1.b1.spca)

summary1(pc1.b3.spca)

summary1(pc1.g13.spca)

#### Summary results from LASSO model

# average size

mean(size.lasso)

# Mean, empirical SE and percent

summary1(b1.lasso)

summary1(b3.lasso)

summary1(g13.lasso)

#### Summary results from PLSR model

colnames(beta.mat.plsr) = featurenames.scenario3

colMeans(beta.mat.plsr)

#### Summary results from CART model

# average size of the tree (number of terminal nodes)

mean(node.cart)

table(node.cart)

# percent of exposure retain in the model

sum(predictor.cart == 1)*100/n

sum(predictor.cart == 2)*100/n

sum(predictor.cart == 3)*100/n

sum(predictor.cart == 4)*100/n

#### Summary results from BMA model

# mean, model-based SE and percent of coefficients

colnames(prob.bma) = colnames(beta.bma) = colnames(se.bma) = featurenames.scenario3

table.bma = cbind(colMeans(beta.bma), colMeans(se.bma), colMeans(prob.bma))

colnames(table.bma) = c("beta", "se", "P")

table.bma

# average size

sum(prob.bma/100)/n

#################### Data generation for Scenarios 4: ######################

################### Time-series study with 10 pollutants ###################

# Calculate parameters of offsets from real data using GLM model

GAM1 = read.csv("Gam1.csv")

DAMAT = as.data.frame(cbind(GAM1$ER_Asthma, GAM1$Temp, GAM1$RH, GAM1$YEAR, GAM1$SEASON, GAM1$Weekday, GAM1$time))

colnames(DAMAT) = c("asthma", "temp", "RH", "year", "season", "DOW", "time")

DAMAT$time = c(1:366,1:365,1:365)

glm.1 = glm(asthma~factor(season)+factor(year)+factor(DOW)+ns(time,8)+ns(temp,4)+ns(RH,3), family=poisson, data=DAMAT)

offset = predict(glm.1) # offset on the log scale

# Partial autocorrelation function (PACF) for 10 exposures up to lag 5 days

a1 = c(0.32, 0.03, 0.06, 0.04, 0.05)

a2 = c(0.53, -0.04, 0.09, 0, 0.02)

a3 = c(0.82, -0.34, -0.02, 0.04, -0.05)

a4 = c(0.33, -0.02, 0.02, -0.01, -0.03)

a5 = c(0.4, 0.05, 0.08, 0.03, -0.02)

a6 = c(0.8, -0.4, -0.02, 0.05, -0.05)

a7 = c(0.34, 0.07, -0.05, 0.02, 0.05)

a8 = c(0.7, -0.2, 0.02, -0.02, 0.01)

a9 = c(0.55, -0.03, 0.08, 0.01, 0.02)

a10= c(0.5, 0.03, -0.04, 0.05, 0.03)

pacf_matrix = cbind(a1,a2,a3,a4,a5,a6,a7,a8,a9,a10)

# Compute the scales of the sum of PACF coefficients

scales = colSums(pacf_matrix)

# mean and covariance matrix of the 10 exposures

meanExpo = rep(1, 10)

Cov1 = matrix(c( 1, 0.6, 0.4, 0.2,

0.6, 1, 0.5, 0.2,

0.4, 0.5, 1, 0.1,

0.2, 0.2, 0.1, 1), nrow=4, byrow=T)

Cov2 = matrix(rep(0,24), nrow=4, byrow=T)

Cov3 = matrix(rep(0,24), nrow=6, byrow=T)

Cov4 = diag(rep(1,6))

varExpo = rbind(cbind(Cov1, Cov2), cbind(Cov3, Cov4))

# mean and covariance matrix of the log of exposure variables

sigma.tilde = matrix(0, nrow=10, ncol=10)

for(i in 1:10) {

for(j in 1:10) {

sigma.tilde[i,j] = log(1+(varExpo[i,j]/(meanExpo[i]*meanExpo[j])))

}

}

mu.tilde = rep(0,10)

for(i in 1:10) {

mu.tilde[i] = log(meanExpo[i])-sigma.tilde[i,i]/2

}

###### Simulation results from 1,000 iterations

n=1000

N=800

# generate empty vectors or matrices for storing simulation results

pc1.b1.spca = c(); pc1.b3.spca = c(); pc1.b6.spca = c(); pc1.b9.spca = c();

pc1.g13.spca = c(); pc1.g16.spca = c(); size.spca = c();

b1.lasso = c(); b3.lasso = c(); b6.lasso = c(); b9.lasso = c();

g13.lasso = c(); g16.lasso = c(); size.lasso = c();

b1.lcr = c(); b3.lcr = c(); b6.lcr = c(); b9.lcr = c(); g13.lcr = c(); g16.lcr = c();

se.b1.lcr = c(); se.b3.lcr = c(); se.b6.lcr = c(); se.b9.lcr = c(); se.g13.lcr = c();

se.g16.lcr = c(); size.lcr = c();

beta.mat.plsr = matrix(nrow=n, ncol=55);

predictor.cart = c(); node.cart = c();

prob.bma = c(); beta.bma = c(); se.bma = c();

for (iter in 1: n) {

# Generate 10 exposures on the first five days as starting points

Expo = rlnorm.rplus(5, mu.tilde, sigma.tilde)

simExpo = matrix(,1000,10)

simExpo[1:5,] = Expo

# Simulate 10 multivariate exposures by autoregressive models on previous 5 days

for (t in 6:1000){

temp = matrix(0,10,5)

for (i in 1:5){ temp[,i] = diag(pacf_matrix[i,])%*%simExpo[t-i,] }

# include only positive values

sim = rep(0,10)

while(min(sim) <= 0){

sim = rowSums(temp)+diag(1-scales)%*%rep(1,10)+mvrnorm(1,rep(0,10),varExpo)

}

simExpo[t,] = sim

}

# Remove data after the burn-in phase

burnin = 200

simExpo = simExpo[(burnin+1):1000,] # total 800 days

# Distribute 10 exposures

X1 = simExpo[,1]

X2 = simExpo[,2]

X3 = simExpo[,3]

X4 = simExpo[,4]

X5 = simExpo[,5]

X6 = simExpo[,6]

X7 = simExpo[,7]

X8 = simExpo[,8]

X9 = simExpo[,9]

X10 = simExpo[,10]

lambda = exp(0.2*X1+0.2*X3+0.2*X6+0.2*X9+0.1*X1*X3+0.1*X1*X6)

# Rescale to keep the mean of mu the same scale of offset

mu = lambda*(exp(offset[1:800])/mean(lambda)

# Generate outcome Y by a two-stage Poission regression

Y = rep(0,800)

for (i in 1:800){ Y[i] = rpois(1,mu[i]) }

log_offset = offset[1:800]-log(mean(lambda))

# Predictor matrix

predictors.scenario4 = matrix(0,800,55)

predictors.scenario4[,1:10] = simExpo

col.count = 11

for (i in 1:9) {

for (j in (i+1):10) {

predictors.scenario4[, col.count] = simExpo[,i]*simExpo[,j];

col.count = col.count+1;

}

}

featurenames.scenario4 = c()

for (i in 1:10) { featurenames.scenario4[i] = paste("X", i, sep = "") }

for (i in 1:9) {

for (j in (i+1):10) {

featurenames.scenario4 = c(featurenames.scenario4, paste("X", i, "_", j, sep = ""))

}

}

colnames(predictors.scenario4) = featurenames.scenario4

######## Method I. Deletion/Substitution/Addition ########

########## Fail to work under time-series study ########

######## Method II. Supervised PCA (SPCA) ########

#### Step 1-5 modified from SPCA by Dr. Steven Roberts ####

# step 1: Fit models containing a single pollutant using training data

wald = rep(0,55)

for (i in 1:55) {

glmfit = glm(Y ~ predictors.scenario4[,i], offset=log_offset, family=poisson);

wald[i] = (summary(glmfit)$coef[,3])[2]

}

# step 2: Sort the variables based on their importance (Wald statistics, large to small)

importance = rev(sort.list(abs(wald)))

# step 3: Find the principal components

# extract only first PC from 90% of the data as the training set

PC_training = matrix(nrow=800*0.9, ncol=55)

PC_training[,1] = predictors.scenario4[1:(800*0.9), sort(importance[1])]

for (i in 2:55) {

PC_training[,i] = predictors.scenario3[1:(800*0.9), sort(importance[1:i])] %*% prcomp(predictors.scenario4[1:(800*0.9), sort(importance[1:i])])$rotation[,1]

}

# extract first PC from 10% of the data as the testing set

PC_testing = matrix(nrow=800*0.1, ncol=55)

PC_testing[,1] = predictors.scenario4[(800*0.9+1):800, sort(importance[1])]

for (i in 2:55) {

PC_testing[,i] = predictors.scenario4[(800*0.9+1):800, sort(importance[1:i])] %*% prcomp(predictors.scenario4[(800*0.9+1):800, sort(importance[1:i])])$rotation[,1]

}

# step 4: Compute test errors

testerror = rep(0, 55)

for (i in 1:55) {

# fit models to the training set

PC = PC_training[,i]

log_mu = log_offset[1:(800*0.9)]

fit = glm(Y[1:(800*0.9)] ~ PC, offset=log_mu, family=poisson)

# find the perdiction error for each model using testing set

test = predict(fit, data.frame(PC=PC_testing[,i], log_mu=log_offset[(800*0.9+1):800]), type="response")

testerror[i] = sum((test-Y[(800*0.9+1):800])^2)

}

# step 5: The model with the lowest prediction error was then re-fitted to the entire dataset

# The number of covariates included into the "best" model

s.spca = which(testerror == min(testerror))

size.spca = c(size.spca, s.spca)

# list names of covariates included into the "best" model

f.spca = featurenames.scenario4[importance[1:s.spca]]

Z.spca = predictors.scenario4[, importance[1:s.spca]] # reduced covariates subset

# The loadings are estimated from the entire data and the model re-fitted

if (s.spca ==1) { PC_best = Z.spca }

if (s.spca >1) { PC_best = Z.spca %*% prcomp(Z.spca)$rotation[,1] }

# apply GLM model

scenario4.spca = glm(Y ~ PC_best, offset=log_offset, family=poisson)

beta.spca = scenario4.spca$coef[[2]] * prcomp(Z.spca)$rotation[,1]

if (s.spca ==1) { names(beta.spca)=featurenames.scenario4[importance[1]] }

# extract beta coefficients

pc1.b1.spca = c(pc1.b1.spca, beta.spca[names(beta.spca) == "X1"])

pc1.b3.spca = c(pc1.b3.spca, beta.spca[names(beta.spca) == "X3"])

pc1.b6.spca = c(pc1.b6.spca, beta.spca[names(beta.spca) == "X6"])

pc1.b9.spca = c(pc1.b9.spca, beta.spca[names(beta.spca) == "X9"])

pc1.g13.spca = c(pc1.g13.spca, beta.spca[names(beta.spca) == "X1_3"])

pc1.g16.spca = c(pc1.g16.spca, beta.spca[names(beta.spca) == "X1_6"])

######## Method III. lasso regression ########

# use glmnet package: alpha=1(lasso), 0(ridge), 0~1(elastic net)

scenario4.lasso = glmnet(predictors.scenario4, Y, family="poisson", offset=log_offset, alpha=1)

scenario4.lasso.cv = cv.glmnet(predictors.scenario4, Y, family="poisson", offset=log_offset, alpha=1)

betas.lasso = coef(scenario4.lasso, s=scenario4.lasso.cv$lambda.min)

# extract LASSO coefficients

b1.lasso = c(b1.lasso, betas.lasso [2])

b3.lasso = c(b3.lasso, betas.lasso [4])

b6.lasso = c(b6.lasso, betas.lasso [7])

b9.lasso = c(b9.lasso, betas.lasso [10])

g13.lasso = c(g13.lasso, betas.lasso [13])

g16.lasso = c(g16.lasso, betas.lasso [16])

# model size (intercept not included)

if (betas.lasso [1] == 0.00000000) { s.lasso = sum(betas.lasso != 0.00000000) }

else { s.lasso = sum(betas.lasso != 0.00000000)-1 }

size.lasso = cbind(size.lasso, s.lasso)

######## Method IV. Partial least square regression (PLSR) ########

data.plsr = data.frame(cbind(Y, predictors.scenario4, log_offset))

# Apply PLSR for glm function

scenario4.plsr = plsRglm(Y~predictors.scenario4, offset=log_offset, data=data.plsr, nt=10, model="pls-glm-poisson")

# use BIC to determine optimal number of components

comp.plsr = which(scenario4.plsr$InfCrit[,2] == min(scenario4.plsr$InfCrit[,2]))-1

# refit model and extract beta coefficients from the optimal model

opt.scenario4.plsr = plsRglm(Y~predictors.scenario4, offset=log_offset, data=data.plsr, nt=comp.plsr, model="pls-glm-poisson")

beta.mat.plsr[iter, ] = opt.scenario4.plsr$Coeffs[2:56]

######## Method V. Classification & regression tree ########

# grow the tree

scenario4.cart = rpart(Y ~ X1+X2+X3+X4+X5+X6+X7+X8+X9+X10, method = "anova")

# prune the tree by min CP

scenario4.pcart = prune(scenario4.cart,

cp=scenario4.cart$cptable[which.min(scenario4.cart$cptable[, "xerror"]), "CP"])

# number of terminal nodes

k.cart = sum(scenario4.pcart$frame$var=="<leaf>")

node.cart = c(node.cart, k.cart)

# extract all unique variables included in CART

j = dim(scenario4.pcart$frame[1])[1]

var = c()

for (i in 1:j)

{ var = c(var, scenario4.pcart$frame[i,1]) }

predictor.cart = c(predictor.cart, unique(var[var!=1]-1))

######## Method VI. Bayesian Model Averaging (BMA) ########

# apply BMA

scenario4.bma = bic.glm(predictors.scenario4, Y, offset=log_offset, maxCol=56 glm.family=poisson(), factor.type=F)

# extract coefficients

prob.bma = rbind(prob.bma, summary(scenario4.bma)[2:56,1])

beta.bma = rbind(beta.bma, summary(scenario4.bma)[2:56,2])

se.bma = rbind(se.bma, summary(scenario4.bma)[2:56,3])

}

#### Summary results from SPCA model

# average size

mean(size.spca)

# Mean, empirical SE and percent

summary1(pc1.b1.spca)

summary1(pc1.b3.spca)

summary1(pc1.b6.spca)

summary1(pc1.b9.spca)

summary1(pc1.g13.spca)

summary1(pc1.g16.spca)

#### Summary results from LASSO model

# average size

mean(size.lasso)

# Mean, empirical SE and percent

summary1(b1.lasso)

summary1(b3.lasso)

summary1(b6.lasso)

summary1(b9.lasso)

summary1(g13.lasso)

summary1(g16.lasso)

#### Summary results from PLSR model

colnames(beta.mat.plsr) = featurenames.scenario4

colMeans(beta.mat.plsr)

#### Summary results from CART model

# average size of the tree (number of terminal nodes)

mean(node.cart)

# percent of exposure retain in the model

sum(predictor.cart == 1)*100/n

sum(predictor.cart == 3)*100/n

sum(predictor.cart == 6)*100/n

sum(predictor.cart == 9)*100/n

#### Summary results from BMA model

# mean, model-based SE and percent of coefficients

colnames(prob.bma) = colnames(beta.bma) = colnames(se.bma) = featurenames.scenario4

table.bma = cbind(colMeans(beta.bma), colMeans(se.bma), colMeans(prob.bma))

colnames(table.bma) = c("beta", "se", "P")

table.bma

# average size

sum(prob.bma/100)/n
