# Supplementary material for: Statistical strategies for constructing health risk models with multiple pollutants and their interactions: possible choices and comparisons
Source: Environ Health. 2013 Oct 4;12:85. doi: 10.1186/1476-069X-12-85 (PMC3857674; doi:10.1186/1476-069X-12-85)
Supplement: Additional file 2 — Supplemental material. [file 1476-069X-12-85-S2.docx]

**Supplemental Material**

**Table 1. Specification of seasonal effects and partial autocorrelation function (PACF) coefficients in simulated time-series studies**

1. **Seasonal effects C_i_ of 4 candidate pollutants**

|  | **X_1_** | **X_2_** | **X_3_** | **X_4_** |
| --- | --- | --- | --- | --- |
| **C_1_** | 1.10 | 2.23 | 1.72 | 0.97 |
| **C_2_** | 1.08 | 1.93 | 1.94 | 0.82 |
| **C_3_** | 1.55 | 2.27 | 1.75 | 1.11 |
| **C_4_** | 1.20 | 2.78 | 2.14 | 1.12 |

1. **PACF coefficients Ф_kj_ on previous 10 days in simulation scenario 3 with 4 candidate pollutants**

|  | **Lag 1** | **Lag 2** | **Lag 3** | **Lag 4** | **Lag 5** | **Lag 6** | **Lag 7** | **Lag 8** | **Lag 9** | **Lag 10** |
| --- | --- | --- | --- | --- | --- | --- | --- | --- | --- | --- |
| **Ф_1j_** | 0.3243 | 0.0310 | 0.0568 | 0.0400 | 0.0146 | 0.0434 | 0.0900 | 0.0386 | 0.0130 | 0.0522 |
| **Ф_2j_** | 0.5287 | -0.0372 | 0.0939 | 0.0022 | 0.0375 | 0.1192 | 0.0445 | 0.0514 | 0.0203 | 0.0235 |
| **Ф_3j_** | 0.8155 | -0.3358 | -0.0184 | 0.0444 | -0.0153 | 0.0465 | -0.0160 | 0.0140 | 0.0444 | -0.0517 |
| **Ф_4j_** | 0.3301 | -0.0174 | 0.0198 | -0.0094 | -0.0266 | 0.0362 | 0.0326 | 0.0305 | -0.0288 | -0.0030 |

1. **PACF coefficients Ф_kj_ on previous 5 days in simulation scenario 4 with 10 candidate pollutants**

|  | **Lag 1** | **Lag 2** | **Lag 3** | **Lag 4** | **Lag 5** |
| --- | --- | --- | --- | --- | --- |
| **Ф_1j_** | 0.32 | 0.03 | 0.06 | 0.04 | 0.05 |
| **Ф_2j_** | 0.53 | -0.04 | 0.09 | 0 | 0.02 |
| **Ф_3j_** | 0.82 | -0.34 | -0.02 | 0.04 | -0.05 |
| **Ф_4j_** | 0.33 | -0.02 | 0.02 | -0.01 | -0.03 |
| **Ф_5j_** | 0.40 | 0.05 | 0.08 | 0.03 | -0.02 |
| **Ф_6j_** | 0.80 | -0.40 | -0.02 | 0.05 | -0.05 |
| **Ф_7j_** | 0.34 | 0.07 | -0.05 | 0.02 | 0.05 |
| **Ф_8j_** | 0.70 | -0.20 | 0.02 | -0.02 | 0.01 |
| **Ф_9j_** | 0.55 | -0.03 | 0.08 | 0.01 | 0.02 |
| **Ф_10j_** | 0.50 | 0.03 | -0.04 | 0.05 | 0.02 |

**Table 2. Distribution of National Health and Nutrition Examination Survey (NHANES) study population by covariates (2005-2008)**

| **Covariate** | **Level** | | **N (percent)*** |
| --- | --- | --- | --- |
| Age | | 12-19 | 944 (25.0%) |
|  |  | 20-34 | 753 (20.0%) |
|  |  | 35-49 | 741 (19.6%) |
|  |  | 50-64 | 688 (18.2%) |
|  |  | 65-79 | 488 (12.9%) |
|  |  | 80+ | 159 (4.2%) |
| Gender | | Male | 1871 (49.6%) |
|  |  | Female | 1902 (50.4%) |
| Race/Ethnicity | | Mexican American | 800 (21.2%) |
|  |  | Other Hispanic | 248 (6.60%) |
|  |  | Non-Hispanic White | 1698 (45.0%) |
|  |  | Non-Hispanic Black | 870 (23.1%) |
|  |  | Other Race/Multi-Racial | 157 (4.20%) |
| Poverty income ratio (PIR) | | 0-0.99 | 812 (21.5%) |
|  |  | 1-1.99 | 984 (26.1%) |
|  |  | 2-2.99 | 583 (15.5%) |
|  |  | 3-3.99 | 450 (11.9%) |
|  |  | 4-5 | 944 (25.0%) |
| BMI (kg/m^2^) | | Underweight (<18.5) | 185 (4.90%) |
|  |  | Normal weight (18.5-24) | 1262 (33.4%) |
|  |  | Overweight (25-29) | 1132 (30.0%) |
|  |  | Obese I (30-34) | 644 (17.1%) |
|  |  | Obese II 35+ | 550 (14.6%) |

* Sample sizes and percents unadjusted for sampling weights.

**Table 3. Pearson correlation coefficients between four groups of exposures from NHANES study (2005-2008)**

**(a) Phthalates**

|  | MEHHP | MEHP | MECPP | MnBP | MiBP | MBzP | MEP | MCPP |
| --- | --- | --- | --- | --- | --- | --- | --- | --- |
| MEOHP | 0.98536* | 0.81658* | 0.95667* | 0.54447* | 0.52374* | 0.52859* | 0.27337* | 0.59584* |
| MEHHP | 1 | 0.82230* | 0.95433* | 0.53583* | 0.52126* | 0.51874* | 0.26925* | 0.59385* |
| MEHP |  | 1 | 0.78788* | 0.38143* | 0.38111* | 0.36291* | 0.21415* | 0.43349* |
| MECPP |  |  | 1 | 0.49884* | 0.48635* | 0.48030* | 0.25963* | 0.59618* |
| MnBP |  |  |  | 1 | 0.74923* | 0.70880* | 0.40139* | 0.60915* |
| MiBP |  |  |  |  | 1 | 0.62461* | 0.37842* | 0.56644* |
| MBzP |  |  |  |  |  | 1 | 0.33354* | 0.55979* |
| MEP |  |  |  |  |  |  | 1 | 0.28092* |

**p<0.05*

**(b) Phenols**

|  | BP3 | OP | TCS | BPAR | EPAR | MPAR | PPAR |
| --- | --- | --- | --- | --- | --- | --- | --- |
| BPA | 0.18428* | 0.09741* | 0.15452* | 0.09382* | 0.11745* | 0.20001* | 0.17284* |
| BP3 | 1 | 0.01205 | 0.15869* | 0.26749* | 0.23744* | 0.28799* | 0.28631* |
| OP |  | 1 | 0.05218* | 0.01437 | 0.00237 | 0.01412 | 0.02352 |
| TCS |  |  | 1 | 0.10362* | 0.10303* | 0.15256* | 0.14034* |
| BPAR |  |  |  | 1 | 0.51478* | 0.46087* | 0.48405* |
| EPAR |  |  |  |  | 1 | 0.42804* | 0.43767* |
| MPAR |  |  |  |  |  | 1 | 0.81628* |

**p<0.05*

**(c) Pesticides**

|  | 2,5-DCP | OPP | 2,4,5-TCP | 2,4,6-TCP |
| --- | --- | --- | --- | --- |
| 2,4-DCP | 0.87198* | 0.07817* | 0.23637* | 0.25995* |
| 2,5-DCP | 1 | 0.07469* | 0.19863* | 0.20625* |
| OPP |  | 1 | 0.06466* | 0.10072* |
| 2,4,5-TCP |  |  | 1 | 0.36657* |

**p<0.05*

**(d) Perchlorate and related anions**

|  | NO3 | SCN |
| --- | --- | --- |
| P8 | 0.54723* | 0.19034* |
| NO3 | 1 | 0.39261* |

**p<0.05*

**Table 4. Estimates of regression coefficients from single-exposure models from NHANES study (2005-2008)**

|  | **Bilirubin** | | | |  | **GGT** | | | |
| --- | --- | --- | --- | --- | --- | --- | --- | --- | --- |
|  | **Estimate** | **SE** | **t value** | **Pr(>\|t\|)** |  | **Estimate** | **SE** | **t value** | **Pr(>\|t\|)** |
| MnBP | -0.025 | 0.008 | -3.270 | 0.001 |  | 0.020 | 0.012 | 1.735 | 0.083 |
| MEP | -0.008 | 0.005 | -1.713 | 0.087 |  | 0.003 | 0.007 | 0.465 | 0.642 |
| MEHP | -0.028 | 0.005 | -5.416 | <0.001 |  | 0.003 | 0.008 | 0.396 | 0.692 |
| MBzP | -0.021 | 0.006 | -3.391 | 0.001 |  | 0.006 | 0.009 | 0.619 | 0.536 |
| MCPP | -0.024 | 0.007 | -3.690 | <0.001 |  | -0.022 | 0.010 | -2.147 | 0.032 |
| MEHHP | -0.030 | 0.005 | -5.678 | <0.001 |  | 0.013 | 0.008 | 1.577 | 0.115 |
| MEOHP | -0.033 | 0.005 | -6.136 | <0.001 |  | -0.019 | 0.008 | -2.308 | 0.021 |
| MiBP | -0.009 | 0.007 | -1.265 | 0.206 |  | 0.032 | 0.011 | 3.017 | 0.003 |
| MECPP | -0.020 | 0.006 | -3.353 | 0.001 |  | <0.001 | 0.009 | -0.042 | 0.967 |
| BPA | -0.007 | 0.007 | -0.903 | 0.367 |  | -0.013 | 0.011 | -1.207 | 0.227 |
| BP3 | 0.006 | 0.003 | 1.898 | 0.058 |  | 0.009 | 0.005 | 1.881 | 0.060 |
| OP | 0.018 | 0.020 | 0.923 | 0.356 |  | -0.015 | 0.030 | -0.503 | 0.615 |
| TCS | <0.001 | 0.003 | -0.086 | 0.932 |  | 0.002 | 0.005 | 0.473 | 0.636 |
| BPAR | <0.001 | 0.004 | 0.046 | 0.963 |  | 0.002 | 0.006 | 0.376 | 0.707 |
| EPAR | 0.015 | 0.004 | 3.627 | <0.001 |  | 0.034 | 0.006 | 5.421 | <0.001 |
| MPAR | -0.001 | 0.004 | -0.273 | 0.785 |  | -0.001 | 0.006 | -0.089 | 0.929 |
| PPAR | -0.001 | 0.003 | -0.169 | 0.865 |  | -0.002 | 0.005 | -0.448 | 0.654 |
| 2,4-DCP | -0.010 | 0.005 | -2.133 | 0.033 |  | -0.015 | 0.007 | -2.093 | 0.036 |
| 2,5-DCP | -0.007 | 0.003 | -2.244 | 0.025 |  | -0.012 | 0.005 | -2.402 | 0.016 |
| OPP | 0.006 | 0.009 | 0.643 | 0.520 |  | 0.001 | 0.014 | 0.044 | 0.965 |
| 2,4,5-TCP | -0.021 | 0.011 | -1.940 | 0.052 |  | -0.017 | 0.017 | -1.040 | 0.298 |
| 2,4,6-TCP | -0.033 | 0.012 | -2.676 | 0.007 |  | -0.022 | 0.019 | -1.179 | 0.238 |
| P8 | -0.068 | 0.008 | -8.416 | <0.001 |  | -0.030 | 0.013 | -2.403 | 0.016 |
| NO3 | -0.045 | 0.011 | -3.974 | <0.001 |  | -0.013 | 0.018 | -0.763 | 0.445 |
| SCN | -0.005 | 0.007 | -0.700 | 0.484 |  | 0.013 | 0.011 | 1.202 | 0.229 |

SE, standard error.

**Figure 5. Pre-screening of exposure variables by correlation analysis and CART for the NHANES data (2005-2008)**

**
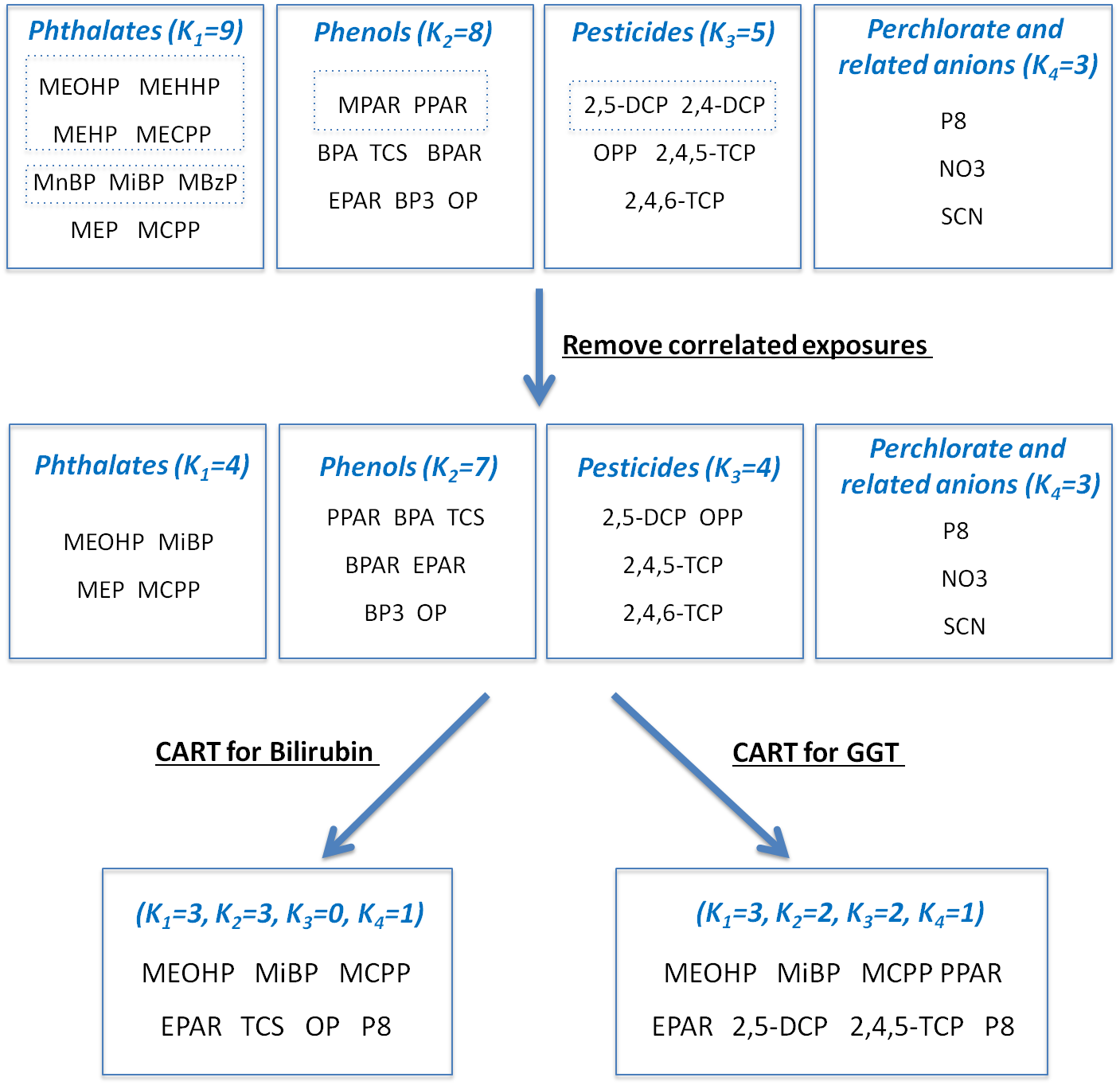
**

Exposures in the same dashed rectangle were highly correlated (Pearson correlation coefficient > 0.60). K_i_ refers to the number of variables retained in the i^th^ group of exposures.

**Table 6. Additional measures of performance for five statistical methods under different simulated scenarios over 1000 replicates**

| **Scenario** | **Measure** | **BMA** | **DSA** | **LASSO** | **PLSR** | **SPCA** |
| --- | --- | --- | --- | --- | --- | --- |
| Scenario 1 | FPR ^1^ | 16.6% | 34.5% | 22.9% | N/A | 64.2% |
|  | TPR ^2^ | 66.9% | 68.1% | 74.0% | N/A | 87.9% |
|  | MSE-CP ^3^ | 0.446 | 0.969 | 0.373 | 0.424 | 0.440 |
|  | MSE-NP ^4^ | 0.044 | 0.381 | 0.266 | 0.025 | 0.049 |
| Scenario 2:  (A) Regression analysis | FPR | N/A | 7.9% | 9.6% | N/A | 35.8% |
|  | TPR | N/A | 57.3% | 46.5% | N/A | 94.4% |
|  | MSE-CP | N/A | 0.868 | 1.005 | 0.955 | 1.005 |
|  | MSE-NP | N/A | 0.993 | 1.028 | 0.099 | 0.062 |
| Scenario 2:  (B) Two-step modeling strategy | FPR | 0.60% | 0.08% | 0.80% | N/A | 1.45% |
|  | TPR | 68.0% | 57.5% | 71.8% | N/A | 75.8% |
|  | MSE-CP | 0.864 | 0.816 | 0.743 | 0.685 | 1.049 |
|  | MSE-NP | 0.103 | 0.0003 | 0.125 | 0.102 | 0.122 |
| Scenario 3 | FPR | 23.3% | N/A | 34.3% | N/A | 2.4% |
|  | TPR | 94.0% | N/A | 100% | N/A | 36.5% |
|  | MSE-CP | 0.061 | N/A | 0.006 | 0.016 | 0.188 |
|  | MSE-NP | 0.003 | N/A | 0.001 | 0.010 | 0.0004 |
| Scenario 4 | FPR | 15.1% | N/A | 30.8% | N/A | 14.2% |
|  | TPR | 95.5% | N/A | 99.9% | N/A | 47.6% |
|  | MSE-CP | 0.040 | N/A | 0.017 | 0.035 | 0.165 |
|  | MSE-NP | 0.021 | N/A | 0.001 | 0.013 | 0.007 |

1: FPR, false positive rate, measures the mean of the ratio that the number of falsely “identified” null predictors divided by the total number of null predictors, where in BMA predictors with their posterior probabilities greater than 10% are regarded as identified, in DSA those selected into the best predic­tive model by cross-validation are viewed as identified, in LASSO estimated regression coefficients not equal to zero are considered identified, and in SPCA predictors are identified if their Wald’s statistics from univariate models are larger than a threshold value. 2: TPR, true positive rate, represents the mean of ratio that the number of correctly identified causal predictors divided by the total number of causal predictors. PLSR does not have a FPR or TPR since it involves no variable selection. 3: MSE-CP, mean squared error for causal predictors. 4: MSE-NP, mean squared error for null predictors.

**Table 7. Estimated effects of exposure variables in proposed models for the NHANES data**

| **Response - Bilirubin** | | | |  | **Response - GGT** | | | |
| --- | --- | --- | --- | --- | --- | --- | --- | --- |
| **Exposure** | **Estimate** | **SE** | **p-value** |  | **Exposure** | **Estimate** | **SE** | **p-value** |
| EPAR | 0.029 | 0.007 | <0.001 |  | EPAR | 0.075 | 0.016 | <0.001 |
| MEOHP | -0.027 | 0.005 | <0.001 |  | P8 | -0.045 | 0.014 | 0.001 |
| P8 | -0.061 | 0.008 | <0.001 |  | 2,4,5-TCP | -0.075 | 0.025 | 0.003 |
| TCS | 0.003 | 0.004 | 0.361 |  | MiBP | 0.031 | 0.012 | 0.009 |
| TCS*EPAR | -0.005 | 0.002 | 0.021 |  | MCPP | -0.039 | 0.016 | 0.015 |
|  |  |  |  |  | PPAR | 0.036 | 0.016 | 0.029 |
|  |  |  |  |  | 2,5-DCP | -0.011 | 0.005 | 0.034 |
|  |  |  |  |  | MEOHP | -0.017 | 0.009 | 0.062 |
|  |  |  |  |  | EPAR*PPAR | -0.015 | 0.003 | <0.001 |
|  |  |  |  |  | EPAR*MCPP | -0.021 | 0.006 | 0.001 |
|  |  |  |  |  | P8*MCPP | 0.027 | 0.008 | 0.001 |
|  |  |  |  |  | EPAR*MiBP | 0.019 | 0.006 | 0.002 |
|  |  |  |  |  | PPAR*2,4,5-TCP | 0.019 | 0.007 | 0.005 |

SE, standard error.

**Table 8. Estimated effects of exposure variables in the proposed model for the DAMAT data**

| **Exposure** | **Estimate** | **Standard error** | **p-value** |
| --- | --- | --- | --- |
| PM_2.5_ | 0.004 | 0.002 | 0.01 |
| CO | 0.069 | 0.034 | 0.04 |
